# Exome sequencing of 85 Williams–Beuren syndrome cases rules out coding variation as a major contributor to remaining variance in social behavior

**DOI:** 10.1002/mgg3.429

**Published:** 2018-07-15

**Authors:** Nathan D. Kopp, Phoebe C. R. Parrish, Michael Lugo, Joseph D. Dougherty, Beth A. Kozel

**Affiliations:** ^1^ Department of Genetics Washington University School of Medicine St. Louis Missouri; ^2^ National Heart Lung and Blood Institute National Institutes of Health Bethesda Maryland; ^3^ Department of Pediatrics Washington University School of Medicine St. Louis Missouri; ^4^ Department of Psychiatry Washington University School of Medicine St. Louis Missouri

**Keywords:** autism spectrum disorder, exome variation, social responsiveness scale, Williams–Beuren syndrome

## Abstract

**Background:**

Large, multigenic deletions at chromosome 7q11.23 result in a highly penetrant constellation of physical and behavioral symptoms known as Williams–Beuren syndrome (WS). Of particular interest is the unusual social‐cognitive profile evidenced by deficits in social cognition and communication reminiscent of autism spectrum disorders (ASD) that are juxtaposed with normal or even relatively enhanced social motivation. Interestingly, duplications in the same region also result in ASD‐like phenotypes as well as social phobias. Thus, the region clearly regulates human social motivation and behavior, yet the relevant gene(s) have not been definitively identified.

**Method:**

Here, we deeply phenotyped 85 individuals with WS and used exome sequencing to analyze common and rare variation for association with the remaining variance in social behavior as assessed by the Social Responsiveness Scale.

**Results:**

We replicated the previously reported unusual juxtaposition of behavioral symptoms in this new patient collection, but we did not find any new alleles of large effect in the targeted analysis of the remaining copy of genes in the Williams syndrome critical region. However, we report on two nominally significant SNPs in two genes that have been implicated in the cognitive and social phenotypes of Williams syndrome, *BAZ1B* and *GTF2IRD1*. Secondary discovery driven explorations focusing on known ASD genes and an exome wide scan do not highlight any variants of a large effect.

**Conclusions:**

Whole exome sequencing of 85 individuals with WS did not support the hypothesis that there are variants of large effect within the remaining Williams syndrome critical region that contribute to the social phenotype. This deeply phenotyped and genotyped patient cohort with a defined mutation provides the opportunity for similar analyses focusing on noncoding variation and/or other phenotypic domains.

## 1 INTRODUCTION

Williams–Beuren syndrome (WS) (OMIM #194050) is a neurodevelopmental disorder caused by a 1.5 to 1.8 Mbp deletion on chromosome 7q11.23. The deletion causes a constellation of symptoms that include cardiovascular pathology, craniofacial dysmorphology, and a unique cognitive and personality profile (Järvinen, Korenberg, & Bellugi, 2013; Mervis et al., 2000; Williams, Barratt‐Boyes, & Lowe, 1961). The well‐defined genetic lesion that causes WS is an opportunity to assess genotype–phenotype correlations. To date, only the cardiovascular phenotype has been convincingly linked to the haploinsufficiency of a single gene—the *ELN* gene (Ewart et al., 1993; Li et al., 1997). Studying rare events that result in atypical deletions sparing different genes in the Williams syndrome critical region (WSCR), as well as single gene knock out studies in mouse models, have suggested that *GTF2IRD1* and *BAZ1B* play a role in the craniofacial abnormalities (Ashe et al., 2008; Tassabehji et al., 2005). Likewise, the genes *STX1A*,* LIMK1*,* CYLN2*,* BAZ1B, GTF2IRD1*, and *GTF2I* (Dai et al., 2009; Fujiwara, Sanada, Kofuji, & Akagawa, 2016; Gao et al., 2010; van Hagen et al., 2007; Hoogenraad et al., 2002; Lalli et al., 2016; Meng et al., 2002; Morris et al., 2003; Sakurai et al., 2011) have been implicated in the cognitive and behavioral phenotypes.

Understanding contributions to social phenotypes in particular for WS may define genes that regulate human social behavior, providing insight not only into WS, but also in other disorders as well as possible modifiers of social behavior in the general population. Deleting one copy of the genes in the WSCR produces the personality profile observed in WS, which consists of prosocial behaviors such as gregariousness, empathy, retained expressive language skills, and low levels of social anxiety, in spite of high anxiety in other domains (Doyle, Bellugi, Korenberg, & Graham, 2004; Gosch & Pankau, 1997; Järvinen et al., 2013; Reilly, Klima, & Bellugi, 1990; Tager‐Flusberg & Sullivan, 2000). Despite the high social motivation of individuals with WS, they exhibit deficits in social cognition and communication (Klein‐Tasman, Li‐Barber, & Magargee, 2010; Klein‐Tasman, Mervis, Lord, & Phillips, 2007; Klein‐Tasman, Phillips, Lord, Mervis, & Gallo, 2009). The Williams syndrome critical region duplication, 7q11.23 duplication syndrome (Dup7) (OMIM#609757), conversely, is characterized by diametric social behaviors to those seen in WS, including separation anxiety, poor eye contact, and language impairment, as well as overlapping phenotypes such as restricted and repetitive behavior and poor social communication (Klein‐Tasman & Mervis, 2018). It has also been shown that the prevalence of ASD in WS and Dup7 is higher than in the general population and the male sex bias for ASD diagnosis is present among individuals with Dup7 (Klein‐Tasman & Mervis, 2018; Richards, Jones, Groves, Moss, & Oliver, 2015). The similarities and differences in the social communication domains of WS and ASD have been described, and suggest that while both disorders show deficits in social communication, the WS group was not as impaired as the ASD group (Klein‐Tasman et al., 2007, 2009). Unlike ASD, there is no sex bias in the frequency of WS and severity of social and cognitive phenotypes are similar across both sexes (Brawn & Porter, 2014; Dykens, 2003).

As in many diseases of haploinsufficiency, within WS there remains considerable variability in expressivity of the phenotypes, despite the very homogeneous genetic cause. It is thought that both genetic background and the environment introduce variation in the expression of a phenotype. The fact that individuals with WS are hemizygous for 26–28 genes has led to the assertion that variation in the remaining allele could contribute to the severity of symptoms in WS (Delio et al., 2013; Merla, Brunetti‐Pierri, Micale, & Fusco, 2010). The presence of only one copy of genes in the WSCR could unmask the effects of recessive alleles in the region that are more difficult to detect in a diploid setting. Indeed, this logic has been applied to investigate the variability in the cardiovascular phenotype. Delio et al., 2013 sequenced the exons that make up the *ELN* gene in a sample of 55 individuals with WS, but found no clear link between severity of phenotype and remaining genetic variation. However, no similar studies have investigated the social profile of WS, in spite of the fact that there is some evidence that common variation in the region can influence social behavior in the general population. For example, variation in the *GTF2I* gene has been associated with the WS cognitive profile, autism, oxytocin reactivity, amygdala activity, and social anxiety (Crespi & Hurd, 2014; Jabbi et al., 2015; Procyshyn, Spence, Read, Watson, & Crespi, 2017). Furthermore, genes outside of the WSCR are also likely to affect aspects of social behavior. In particular genes that are associated with ASD have a profound effect on social interaction and could harbor variants that modify the phenotype of individuals with WS.

Here, we employ whole exome sequencing to understand how genetic variation within the WSCR, and other protein coding genes, impacts the severity of the WS social phenotype. We generate a rich catalog of genetic variants identified from 85 individuals with the typical WS deletions; each individual has also been assessed with the Social Responsiveness Scale‐2 (SRS) questionnaire, a quantitative measure of reciprocal social behavior. The SRS was first developed to quantify autistic traits in both the general and clinical populations (Constantino & Todd, 2003; Moreno‐De‐Luca et al., 2015). SRS scores have also been used to describe different aspects of the social phenotype in WS (Klein‐Tasman et al., 2010). We then employ a three‐tiered approach to screen for the existence of alleles that contribute to SRS scores in the context of a potentially sensitizing WSCR deletion, ordering the analyses to conserve statistical power. First, we describe the genetic variants observed in the remaining WSCR and test if they can explain the variance in the SRS scores. We find little evidence that these common or rare variants in the region are associated with SRS scores. Next, we go beyond the WSCR and test variants in 71 genes known to be associated with ASD (Sanders et al., 2015), reasoning variation that contributes to autistic features in non‐WS children may modify autistic features in the WS cohort as well. Finally, we test variants throughout the whole exome. We find no genetic variants of sufficient effect size to support the hypothesis that they contribute to the social phenotype in this sample of individuals with WS. However, we have more thoroughly described the variation in the WSCR region as it relates to social behavior and provide the largest genetic dataset to date of individuals with typical WS deletions for future analyses of other phenotypic domains.

## 2 MATERIALS AND METHODS

### 2.1 Ethical compliance and samples

This study was conducted with approval of the IRBs at Washington University School of Medicine and the National Institutes of Health. Consent was obtained prior to inclusion in the study. Once enrolled, participants provided a DNA sample by blood or saliva and their care‐givers filled out health related questionnaires. The 85 individuals that make up our sample have ages that range from 2.5 to 65.5 years with a mean of 16.1 years. Caregivers provided a self‐reported ethnicity. The majority of the sample was reported as white (77 individuals). There are two individuals that are African American, three Chinese, and three others.

### 2.2 Confirmation of diagnosis

WS diagnosis and typical deletion size was confirmed using either chromosomal microarray or quantitative PCR. In some cases, clinical microarray results were derived from the medical record. Array type varied by individual. For the remaining individuals, some received a research array (Cytoscan HD, Applied Biosystems) with analysis using the accompanying ChAS software. Others underwent deletion size assessment using quantitative PCR for genes within and outside of the Williams region using Taqman copy number probes (Thermo‐Fisher, AUTS2: Hs04984177_cn, CALN1: Hs04946916_cn, FZD9: Hs03649975_cn, CLIP2: Hs00899301_cn, HIP1: Hs00052426_cn, POM121C: Hs07529820_cn). Copy number analysis was done according to the manufacturer's instructions and output data analyzed using their Copy Caller software. All individuals were confirmed to have deletions that included the WSCR genes ELN, FZD9 and CLIP2, but did not include genes external to the typical deletion such as CALN, AUTS2, POM121C or HIP1 (data not shown).

### 2.3 Social responsiveness scale

The social responsiveness scale‐2 (SRS) is a 65‐item questionnaire that measures aspects of social interaction that make up the core symptoms of autism spectrum disorders. The output is a total raw score as well as a *T*‐score that is adjusted for sex, age, and the relationship of the reporter to the proband. The total score is made up of the scores of five subcategories that are impaired in ASDs: social awareness (AWR), social cognition (COG), social motivation (MOT), social communication (COM), and behaviors typical of autism such as restricted interests and repetitive behaviors (RRB). The response to each question ranges from 1 (not true) to 4 (almost always true). The *T*‐scores are binned into four groups: normal <59, mild between 60 and 65, moderate between 66 and 75, and severe >76. For this study, the age‐specific (preschool, school age, or adult) SRS‐2 was completed by the participant's caregiver and analyzed as a *T*‐score that is adjusted for sex, age, and the relationship of the reporter. We provide values from the general population that have been previously reported for comparison (Constantino & Todd, 2003; Constantino et al., 2003).

### 2.4 Sequencing and variant calling

Whole exome sequencing and alignment was performed at Washington University in St. Louis by the McDonnell Genome Institute on 85 DNA samples from individuals with WS. Exomes were captured using Nimblegen SeqCap EZ Choice HGSC Library version 2.1, which targets 45.1 Mbp covering 23,585 genes and 189,028 nonoverlapping exons. Exomes were aligned to the GRCh37‐lite genome using bwa –mem v0.7.10 (Li & Durbin, 2009) default settings, samtools v0.1.19 (Li et al., 2009) was used to assign mate pairings, sort, and index the bam files. Duplicates were marked using Picard MarkDuplicates v1.113.

Variant calling was done following GATK best practices on the aligned exomes (DePristo et al., 2011). Briefly, using GATK v3.6.0 indels were realigned and the base quality scores recalibrated. Variants were initially called per sample using the haplotype caller tool, followed by jointly calling variants. To improve variant calls, we recalibrated variants and used a truth sensitivity tranche of 97 for SNPs, and a truth sensitivity tranche of 94 for indels. These thresholds were chosen to maximize the number of known and novel variants while still being stringent enough to limit the number of false positive variant calls. To further filter the variants we used the VariantFiltration tool to filter variant sites that had lower than a 10× average coverage or an inbreeding coefficient < −0.20 to remove sites with excess heterozygosity. Genotype calls were filtered and considered to be missing if they had a genotype quality score of <20, which refers to a 99% probability that the call is correct. Finally, using vcftools v0.1.14 (Danecek et al., 2011), we removed sites that had a genotype missing rate of >10%, as well as sites that no longer showed any variation. This produced a call set of 202,820 variant sites. The final call set has a Ti/Tv ratio of 2.76 and a dbSNP rate of 88.5%. These metrics are consistent with quality variant calls and a low false positive rate.

### 2.5 Variant annotation

The variant call set was split into three groups using vcftools: (1) variants in the Williams syndrome critical region (WSCR) defined by hg19 coordinates chr7:72,395,660–74,267,841 (2) variants located in 71 genes associated with ASD (Sanders et al., 2015), and (3) the remaining nonoverlapping variants. All sets include exonic variants as well as variants located in introns that are pulled down by the capture reagents. Bcftools v1.2 (Li et al., 2009) was used to split multiallelic sites into separate lines for each allele and left normalized so positions would be compatible with ANNOVAR annotation files version 2016‐02‐01 (Wang, Li, & Hakonarson, 2010). The ANNOVAR table_annovar.pl function was used to annotate all three variant call sets with the RefSeq gene annotation, variant consequence, ExAC allele frequency (Lek et al., 2016), sample specific allele frequency, dbsnp147 name, clinical significance assessed by ClinVar (Landrum et al., 2016). Missense variants were also annotated with measures of deleteriousness compiled in dbNSFPv3.3a (Liu, Wu, Li, & Boerwinkle, 2016). We highlight the CADD PHRED score and MetaLR as two measures of variant deleteriousness. CADD scores are defined at each base in the genome and for every possible single‐nucleotide change (Kircher et al., 2014). CADD scores compare 65 annotations, including functional data as well as conservation scores, between fixed human derived alleles and simulated variants. Deleterious variants should be depleted in the observed fixed alleles and not in the simulated variants. CADD PHRED scores represent the relative rank of a CADD score compared to all other possible allele CADD scores; a CADD score of 10 means this allele is ranked as the top 10% of all possible CADD scores. Larger CADD PHRED score indicates an increased predication of deleteriousness. MetaLR uses logistic regression to incorporate information from nine other variant annotations that consider function as well as conservation (Dong et al., 2015). The model was trained on true deleterious variants and true neutral variants described in the Uniprot database. The composite MetaLR score was found to have greater predictive ability than any of the single scores that make up MetaLR.

### 2.6 Power analysis

We performed a power analysis to provide the limits of genetic effects that we would be able to detect given our cohort size. For future studies we also calculate the sample sizes that would be needed to detect different magnitudes of genetic effects. We used the Genetic Power Calculator (Purcell, Cherny, & Sham, 2003). We calculated the predicted power of the current sample size *n* = 85 using a *p*‐value threshold corresponding to the Bonferroni corrected alpha for each set of analyses (WSCR 34 variants, alpha = 0.00147, ASD 381 variants, alpha = 0.000131, WEX 66620 variants, alpha = 7.5 × 10^−7^. Our main hypothesis is variants on the remaining WSCR allele affect the social phenotype; we wanted to calculate the sample sizes that would be required to detect different size genetic effects in the WSCR at different levels of power. We again used the alpha threshold based on the 34 common variants we identified in the exons of the WSCR and report the sample size required to achieve a specific power.

### 2.7 Association analyses

#### 2.7.1 Common variant analysis

The variant call files were converted to plink binary bed format using the GATK tool VariantToBinaryPed. We used PLINK v1.9 (Purcell et al., 2007) –linear option to conduct a quantitative trait association using the SRS *T*‐score as the quantitative trait. Ancestry was controlled for by including the first four principle components, determined by the –pca function in PLINK, as covariates along with sex and age. We used alleles that had a minor allele frequency (MAF) of 0.05 or greater. We performed the association analyses on the three separate groups of variants described in the previous section. It should be noted that allele frequency in the Williams syndrome critical region is inflated because of the hemizygous state of the region in individuals with WS. A MAF of 0.05 in this region corresponds to an allele count of four. In all cases we report the effect size of a variant under an additive model. Though the small sample size of this study limits power, in an exploratory fashion we also performed the same quantitative trait analysis on each of the subscores of the SRS using variants in the WSCR, ASD genes, and the whole exome.

#### 2.7.2 SKAT‐O

SKAT‐O (Lee, Wu, & Lin, 2012)was implemented in the R v3.1.3 environment. SKAT‐O fits a multiple linear regression of all SNPs located in a user provided region. The framework in SKAT‐O allows for correlation between SNPs in a region, where if all SNPs are perfectly correlated this would become a burden test, but also allows SNPs in the same region to have effects in opposite directions. Significance is assessed by region rather than by SNP. We considered each gene that harbors a variant in the WSCR as a separate region for a total of 26 regions. To test for an overall effect of variants in the ASD genes we collapsed the 61 autosomal genes into one region. We used the beta function shape parameters (1,50) to put more weight on SNPs that have lower minor allele frequency, reasoning that rare causal alleles potentially have a greater effect size. We again controlled for age, sex, and the first four principal components.

#### 2.7.3 Polygenic risk score

Polygenic Risk Scores (PRS) can be used to test if there is a contribution of many loci of small effect on the phenotype of interest by summing the effects of variants that may have not reached genome‐wide significance. For a discovery set, we used the publically available summary statistics from the most recent Psychiatric Genome Consortium genome‐wide association study (GWAS) of autism spectrum disorder (The Autism Spectrum Disorders Working Group of the Psychiatric Genomics Consortium 2017), reasoning that genetic risk for autism would contribute to SRS scores. The best‐fit PRS was determined using the high‐resolution functionality in the PRSice software (Euesden, Lewis, & O'Reilly, 2015). All the variants identified throughout the exome with a MAF >0.05 and that are also present the in the discovery set were used to calculate the PRS. Sex, age, and the first four PCs were included as covariates. After clumping there were a total of 23,191 variants used to calculate the PRS. PRSice was used to calculate the significance of the PRS at the best‐fit *p*‐value threshold using 10000 permutation to determine an empirical *p*‐value. PRS for each of the samples was calculated for the total SRS *T*‐score as well as the subscores.

### 2.8 Other statistical analyses

All remaining statistical tests were done in the R v3.1.3 environment. Two sample *t*‐tests were used to compare the means of two groups. ANOVA was used to test differences in mean of subscales of SRS. TukeyHSD post hoc comparison was performed using the multcomp package. The qqman (Turner, 2014) package was used to generate manhattan and qq plots.

## 3 RESULTS

### 3.1 SRS variability in Williams syndrome

The unique social profile of Williams syndrome includes increased social motivation (e.g., indiscriminate approach to strangers), strong eye contact, use of affective language, emotional sensitivity as well as poor social judgment and restricted interests (Doyle et al., 2004; Gosch & Pankau, 1997; Klein‐Tasman & Mervis, 2003; Klein‐Tasman et al., 2007; Reilly et al., 1990; Tager‐Flusberg & Sullivan, 2000). Many comorbidities, such as specific phobias, ADHD, and anxiety, have been commonly reported in WS as well (Dykens, 2003; Einfeld, Tonge, & Florio, 1997; Einfeld, Tonge, & Rees, 2001; Leyfer, Woodruff‐Borden, Klein‐Tasman, Fricke, & Mervis, 2006; Switaj, 2001). To quantify social features in our WS cohort, we used a standard instrument for assessing social reciprocity, parent‐reported SRS scores from 85 individuals with WS.

**Figure 1:**
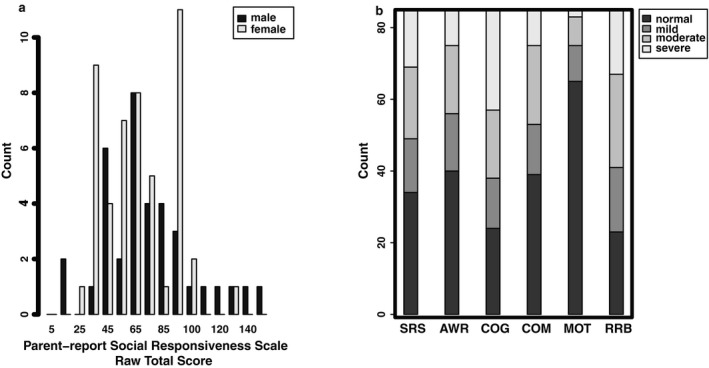
Distribution of Social Responsiveness in 85 individuals with typical WS deletion. (a) Distribution of the raw SRS scores. (b) Severity bins of SRS and subcategory scores

We examined the SRS and its subscores in depth. In our sample, the SRS *T*‐scores are continuously distributed in the WS population with a male mean *T*‐score ± *SD* of 64.58 ± 12.28 (mean male raw score ± *SD* 74.53 ± 32.03) and female mean *T*‐score ± *SD* of 62.94 ± 11.04 (mean female raw score ± *SD* 67.08 ± 26.04) (Figure 1). There is no significant difference in SRS *T*‐scores (*t*_70.76 _= 0.6365, *p* = 0.52) or raw scores (*t*_65.907 _= 1.1445, *p* = 0.257) between sexes. To benchmark the WS values, Constantino & Todd, 2003 measured raw SRS scores in 788 twin pairs from the general population ranging in ages between 7 and 15 and estimated the mean male raw score ± *SD* as 35.3 ± 22.0 and the female mean raw score ± *SD* as 27.5 ± 18.4; males and females were significantly different. In our analysis, we show that individuals with WS have SRS scores that are shifted toward the more impaired end of the spectrum, and we do not detect any significant sex differences in WS, which has been observed in the general population.

Our results largely replicate the results seen in Klein‐Tasman et al., 2010;. The overall *T*‐score distribution reveals that 40% of our samples fall into the no clinically significant impairment range, followed by 41.1% with mild to moderate deficits, and 18.9% with severe deficits. The number of individuals showing no clinical signs in our sample is higher than the 13.4% observed when the parents completed the SRS in Klein‐Tasman et al., 2010; but more similar to the teacher reported results of 38.8%. The subscores also follow a similar pattern to what has been reported previously (Klein‐Tasman et al., 2010). There is a significant effect of subscale on the *T*‐scores (*F*_4,420_ = 24.759, *p* < 0.001) (Figure 1b). Post hoc Tukey all‐pairwise comparisons show that social motivation has significantly better *T*‐scores than all other subscales, consistent with Klein‐Tasman et al., 2010. The social awareness and communication scales are not different from each other, but both show less impairment than social cognition and restricted and repetitive behaviors. Social cognition and restricted and repetitive behaviors were significantly more impaired than all other subscales, but not each other.

The distribution of SRS scores in WS points to the possibility of additional genetic variants that modify the social phenotype. First, we see a larger standard deviation in the SRS data in our sample compared to that of the norming population from Constantino & Todd, 2003. The extra variance suggests individuals with WS are more sensitive to genetic or environmental factors that modify social behavior. Second, in our sample there are only two individuals that show severe social motivation deficits, and these individuals also show severe deficits in the total SRS *T*‐score as well as all other subscales. These outliers also suggest some individuals may harbor additional rare variants of large effect size resulting in a phenotype that is more frankly autistic. To test these two hypotheses, we generated and analyzed exome sequences from this cohort of WS patients.

### 3.2 Identification of variants in the Williams syndrome critical region

Williams syndrome individuals are hemizygous for 1.5–1.8 Mbp on chromosome 7q11.23. Since they only have one remaining allele, our primary hypothesis was that second hits in genes believed to impact social phenotypes within the WSCR would produce more extreme social phenotypes. We performed whole exome sequencing on 85 individuals, all whom have an SRS score. We called 120 variants in the remaining WSCR and annotated them with the allele frequency in our sample, ExAC allele frequency, mutation consequence, clinical significance as assessed by ClinVar, and scores that assess deleteriousness of missense variants cataloged in dbNSFP. (Supporting Information Table S1). Table 1 shows the 55 exonic variants discovered in the region. For display purposes we have only included the CADD PHRED score and the MetaLR score, which is a composite score that incorporates information from nine other measures of deleteriousness and has been shown to have more predictive power than the individual component scores (Dong et al., 2015).

**Table 1:** Annotation of 55 exonic variants discovered in the WSCR

Chr	Start	avsnp147^a^	Alt	MAF	Transcript	Gene	Consequence	MetaLR score	MetaLR prediction^b^	CADD PHRED
7	72413057	rs782618986	A	0.005882	NM_172020	*POM121*	p.S577N	0.011	T	0.006
7	72717686	rs145622470	T	0.01176	NM_001168347	*NSUN5*	p.P399P	.	.	8.726
7	72719048	rs34913552	A	0.01176	NM_001168347	*NSUN5*	p.P183S	0	T	0.002
7	72738534	rs371073794	T	0.01176	NM_001281450	*TRIM50*	p.P84P	.	.	15.11
7	72738561	rs61741334	T	0.04706	NM_001281450	*TRIM50*	p.I75I	.	.	11.01
7	72738762	rs6980258	T	0.9882	NM_001281450	*TRIM50*	p.L8L	.	.	0.46
7	72738763	rs6980124	G	0.9882	NM_001281450	*TRIM50*	p.L8P	.	.	0.001
7	72744246	rs200493820	T	0.01176	NM_001281304	*FKBP6*	p.T90M	0.492	T	13.74
7	72754645	rs56301507	A	0.01176	NM_001281304	*FKBP6*	p.L168L	.	.	3.802
7	72856676	rs1178978	T	0.01176	NM_032408	*BAZ1B*	p.Q1434Q	.	.	11.69
7	72857130	rs150115317	T	0.01176	NM_032408	*BAZ1B*	p.R1340K	0.025	T	23.6
7	72891754	rs2074754	T	0.4	NM_032408	*BAZ1B*	p.S679S	.	.	10.13
7	72936183	.	A	0.01176	NM_032408	*BAZ1B*	p.H27H	.	.	2.032
7	72951640	rs142166738	G	0.01176	NM_001197244	*BCL7B*	p.A142A	.	.	7.437
7	72985148	rs35607697	T	0.03529	NM_012453	*TBL2*	p.V345I	0.014	T	26.3
7	72987758	.	C	0.01176	NM_012453	*TBL2*	p.F164V	0.154	T	27.3
7	72992858	rs76029572	G	0.07059	NM_012453	*TBL2*	p.E8Q	0.054	T	9.196
7	73010754	rs61738649	T	0.05882	NM_032951	*MLXIPL*	p.L626L	.	.	2.706
7	73013901	rs13235543	T	0.1294	NM_032951	*MLXIPL*	p.P342P	.	.	6.53
7	73020301	rs799157	C	0.9647	NM_032951	*MLXIPL*	p.S253S	.	.	2.151
7	73020337	rs3812316	G	0.1059	NM_032951	*MLXIPL*	p.Q241H	0.001	T	19.07
7	73020439	rs12539160	T	0.01176	NM_032951	*MLXIPL*	p.A207A	.	.	12.68
7	73083889	rs61743139	T	0.02353	NM_001077621	*VPS37D*	p.A93A	.	.	18.4
7	73097082	rs79849491	G	0.02353	NM_032317	*DNAJC30*	p.F224F	.	.	0.66
7	73097238	rs1569062	A	0.3294	NM_032317	*DNAJC30*	p.Y172Y	.	.	11.69
7	73122977	rs2229854	A	0.05882	NM_001165903	*STX1A*	p.N50N	.	.	11.25
7	73150934	rs138932141	A	0.01176	NM_001145364	*ABHD11*	p.D244D	.	.	1.115
7	73245591	rs142910620	T	0.01176	NM_001305	*CLDN4*	p.A20A	.	.	17.87
7	73254812	rs13241921	C	0.7882	NM_152559	*WBSCR27*	p.Q107R	0	T	0.001
7	73275565	rs11770052	A	0.7647	NM_182504	*WBSCR28*	p.I14N	0	T	15.45
7	73279361	rs61742124	T	0.1294	NM_182504	*WBSCR28*	p.L37L	.	.	14.82
7	73279413	rs118088869	T	0.03529	NM_182504	*WBSCR28*	p.R55W	0.01	T	15.49
7	73280020	rs1136647	T	0.7176	NM_182504	*WBSCR28*	p.T205T	.	.	12.3
7	73466285	rs6979788	G	0.01176	NM_001278913	*ELN*	p.A271A	.	.	1.511
7	73470714	rs2071307	A	0.4706	NM_001278913	*ELN*	p.G412S	0	T	6.674
7	73474268	rs200512332	T	0.01176	NM_001278913	*ELN*	p.V408V	.	.	9.149
7	73474367	rs61734584	A	0.01176	NM_001278913	*ELN*	p.G441G	.	.	1.008
7	73474825	rs17855988	C	0.07059	NM_001278913	*ELN*	p.G500R	0.007	T	23.2
7	73477524	rs140425210	A	0.01176	NM_001278913	*ELN*	p.G529S	0.131	T	23.7
7	73631177	rs144269935	G	0.02353	NM_014146	*LAT2*	p.I39M	0.013	T	25.9
7	73651743	rs3135688	C	0.01176	NM_001278792	*RFC2*	p.V160V	.	.	8.01
7	73663362	rs1805395	C	0.05882	NM_001278791	*RFC2*	p.E3E	.	.	7.454
7	73731906	rs148561130	T	0.02353	NM_003388	*CLIP2*	p.P10P	.	.	18.78
7	73811479	rs76865959	C	0.01176	NM_003388	*CLIP2*	p.R897R	.	.	4.969
7	73814702	rs17145468	A	0.03529	NM_003388	*CLIP2*	p.D926E	0.006	T	17.3
7	73814749	rs2522943	C	0.9647	NM_003388	*CLIP2*	p.R942P	0	T	18.33
7	73929826	rs111256098	T	0.01176	NM_001199207	*GTF2IRD1*	p.G139G	.	.	12.93
7	73932488	rs112098981	G	0.01176	NM_001199207	*GTF2IRD1*	p.A179A	.	.	9.272
7	73932494	rs145535993	T	0.02353	NM_001199207	*GTF2IRD1*	p.V181V	.	.	10.27
7	73932560	rs17851629	G	0.2118	NM_001199207	*GTF2IRD1*	p.E203E	.	.	9.058
7	73933793	rs148463467	T	0.01176	NM_001199207	*GTF2IRD1*	p.V252V	.	.	14.93
7	73944095	rs61744518	T	0.02353	NM_001199207	*GTF2IRD1*	p.P406P	.	.	16.53
7	73944185	rs2240357	C	0.2353	NM_001199207	*GTF2IRD1*	p.Y436Y	.	.	0.434
7	73953017	rs55634982	T	0.01176	NM_001199207	*GTF2IRD1*	p.S517S	.	.	14.02
7	74211576	rs587728502	C	0.01176	NM_173537	*GTF2IRD2*	p.M759V	0.021	T	0.893

*Notes*

^a^“.” Refers to information that is not applicable; ^b^“T” the missense mutation is predicted to be Tolerated.

We first examined this set of variants to determine if any loss‐of‐function variants might be present in individuals with particularly severe SRS scores in our sample. Upon inspection of the exonic variants, we notice no severe likely protein‐truncating variants. As homozygous nulls for at least two genes in this region (*ELN* and *GTF2I*) are expected to be lethal (Li et al., 1998; Sakurai et al., 2011), we also assessed missense mutations in these genes that might alter function. Based upon the predictions of MetaLR all the missense mutations called are expected to be tolerated. None of the variants were reported as pathogenic in ClinVar. The highest CADD scores observed are a novel variant and SNP rs35607697, both located in the *TBL2* gene. Another novel variant was identified as a synonymous change in the *BAZ1B* gene. Similar results are found for nonexonic variants in the region (Supporting Information Table S1). This suggests that beyond the reduced copy number of the entire WSCR, neither a second rare deleterious coding variant nor any common missense mutations in the WSCR explain individuals with outlier SRS scores. It should be noted that we did not identify any variants in *GTF2I*, one of the primary candidates for mediating the social‐cognitive profile.

### 3.3 Association analyses

**Figure 2:**
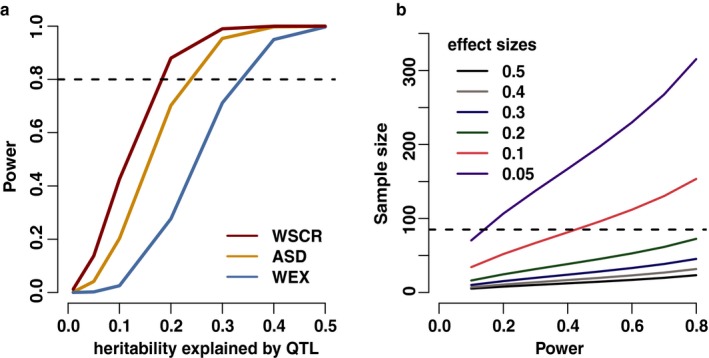
Power analysis. (a) The power to detect variants of different effect sizes for this study. The alpha for the three different sets of analyses was determined by using the Bonferroni correction based on the number of SNPs tested in each analysis. (WSCR: variants in the WSCR, ASD: variants in the 71 ASD genes, WEX: all remaining variants exome wide). (b) The predicted sample sizes that would be required to achieve different levels of power for detecting variants of different effect sizes. The sample size predictions were only done using the alpha for the number of SNPs tested in the WSCR. The horizontal dashed line indicates the sample size of this study

To test the hypothesis that individual variants in the WSCR can explain the variance in the SRS scores in our sample, we perform classic quantitative trait loci associations. Rare disease populations by definition will have small sample sizes such as in this study. We calculated the power of our study to be able to detect variants with different effect sizes and also calculated the number of samples that would be needed to reach a certain power given an effect size (Figure 2). We calculated the power for analyzing variants in the WSCR, variants in 71 ASD genes, and the remaining variants identified throughout the exome. Since we are conducting fewer tests in the WSCR, we have the most power in this analysis, however we are still only powered to detect very large effect sizes that might be unmasked by the hemizygosity of the region, such variants would need to explain more than 10% of the heritability of the trait to achieve 80% power. Most effect sizes for common variants in diploid regions of the genome typically assessed by GWAS for complex traits explain around 1% of the heritability of the trait (Manolio et al., 2009). In order to be able to detect variants that explain 5% of the variance of the trait with 80% power using only variants in the WSCR would require 312 individuals (Figure 2b).

**Figure 3:**
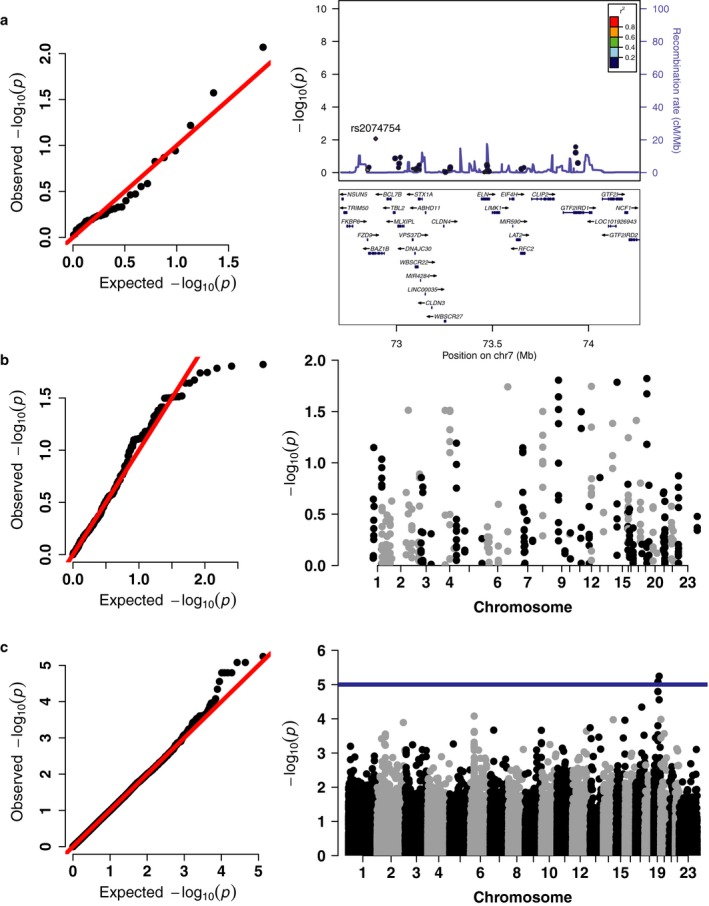
Variants in the WSCR, ASD genes, or whole exome do not contribute to SRS variability in a sample of WS with typical deletions. (a) qq plot showing distribution of *p*‐values for common variants in the WSCR. Locus zoom plot showing the SNPs tested in the WSCR, highlighting the most nominally significant SNP in *BAZ1B*. (b) qq and manhattan plot for variants called in 71 genes associate with ASD from Sanders et al. (2015). (c) qq and manhattan plot for variants exome wide. Blue line demarcates a suggestive *p* value threshold of 1 × 10^−5^

We then performed a quantitative trait association analysis of common variants in the WSCR on the SRS *T*‐scores from the whole cohort. We used PLINK to test for an association on each of the 34 common variants in the WSCR, defined as MAF > 0.05, which corresponds to an allele count of at least four in the WSCR due to the hemizygosity of the region. We adjusted for age, sex, and ancestry. We found no association between any SNP and SRS that survived multiple comparison corrections (Figure 3a). The top five SNPs are displayed in Table 2. Interestingly, the most significant SNP, rs2074754, is located in the *BAZ1B* gene, which has been previously implicated in contributing to the cognitive phenotypes in WS (Lalli et al., 2016). Furthermore, the next most nominally significant SNP is rs61438591, an intronic variant in the *GTIF2RD1* gene, another gene highly implicated in the cognitive and social phenotypes seen in WS (van Hagen et al., 2007; Howard et al., 2012; Schneider et al., 2012; Young et al., 2008).

**Table 2:** Top five SNPs from quantitative trait locus associations

SNP	Alt allele	MAF	Transcript^a^	Gene	Consequence	Beta	95% Confidence interval	Raw p‐value	FDR	Analysis group^b^
rs2074754	T	0.4	NM_032408	*BAZ1B*	p.S679S	3.429	0.9415 –5.917	0.0085	0.2899	WSCR
rs61438591	C	0.2	.	*GTF2IRD1*	Intronic	3.506	0.4648–6.547	0.0267	0.4542	WSCR
rs17851629	G	0.22	NM_016328	*GTF2IRD1*	p.E171E	2.932	−0.0839 to 5.948	0.0605	0.6851	WSCR
rs3812316	G	0.11	NM_032951	*MLXIPL*	p.Q241H	3.402	−0.7692 to 7.572	0.1141	0.8466	WSCR
rs76029572	G	0.07	NM_012453	*TBL2*	p.E8Q	−3.735	−8.587 to 1.117	0.1367	0.8466	WSCR
rs12983010	G	0.07	NM_14469	*CAPN12*	p.C287R	9.286	1.96–16.58	0.0151	0.6587	ASD
rs12553775	A	0.11	.	*PHF2*	Intronic	7.573	1.567–13.58	0.0157	0.6587	ASD
rs140682	C	0.48	NM_000810	*GABRA5*	p.V202V	−4.377	−7.874 to 0.8801	0.0164	0.6587	ASD
rs1805482	A	0.35	NM_000834	*GRIN2B*	p.S555S	4.918	0.9301–8.906	0.0180	0.6587	ASD
rs112318565	G	0.06	.	*ARID1B*	Intronic	10.22	1.918–18.51	0.0182	0.6587	ASD
rs527221	C	0.11	NM_001288765	*DMPK*	p.L334V	13.78	8.246–19.31	5.70 × 10^−6^	0.1522	WEX
rs2546028	C	0.54	.	*ZNF792*	UTR5	−6.95	−9.801 to −4.099	8.32 × 10^−6^	0.1522	WEX
rs2546029	G	0.54	.	*ZNF792*	UTR5	−6.95	−9.801 to −4.099	8.32 × 10^−6^	0.1522	WEX
rs1811	G	0.46	NM_001099437	*ZNF30*	p.Q124R	7.166	4.116–10.22	1.60 × 10^−6^	0.1522	WEX
rs2651109	C	0.46	NM_001099437	*ZNF30*	p.S215S	7.166	4.116–10.22	1.60 × 10^−6^	0.1522	WEX

*Notes*

^a^“.” Refers to information that is not applicable; ^b^WSCR (Williams syndrome critical Region), ASD (71 genes associated with ASD), WEX (variants across Whole Exome).

As the common variants in WSCR showed no association, we wanted to test for the possibility that rare variants could contribute to the variability in SRS *T*‐scores. To test this, we used SKAT‐O, which tests all variants in the region at once and weights each variant by its minor allele frequency. Similarly, we included age, sex, and ancestry as covariates. We tested each gene in the WSCR independently, because we hypothesized only certain genes in the region, such as *STX1A*,* LIMK1*,* CYLN2*,* BAZ1B, GTF2IRD1* (Dai et al., 2009; Fujiwara et al., 2016; Gao et al., 2010; van Hagen et al., 2007; Hoogenraad et al., 2002; Lalli et al., 2016; Meng et al., 2002; Morris et al., 2003; Sakurai et al., 2011) that have been implicated in the cognitive phenotypes would contribute to the social phenotype rather than the entire region. While no gene *p*‐value survives multiple testing corrections, the *ELN* gene has the most nominally significant *p*‐value of 0.013.

The results of our analysis of variation in the WSCR suggest that common and rare variants in the remaining allele do not strongly influence social behavior in WS. This does not exclude the possibility that a second deleterious hit or common variation in other genes outside the region contributes to the variation in the SRS *T*‐scores. To test this, we next examined variation in 71 genes known to be associated with autism spectrum disorders (Sanders et al., 2015). These genes should be enriched for loci that affect social behavior and genetic variation in these genes could contribute to variability seen in WS. We called 1,367 variants in the 71 genes (Supporting Information Table S2). We annotated the variants as above, with clinical significance and measures of deleteriousness compiled in dbNSFP. There are 313 (22.9%) variants that had at least one submission to ClinVar. None of these variants had previous evidence to support pathogenicity. There are 33 missense variants predicted to be deleterious by MetaLR that are seen in 36 individuals in our sample. Despite having a putatively deleterious variant the distribution of SRS *T*‐scores is similar between individuals either carrying or lacking deleterious variants in these genes (*t*_82.999 _= 0.6878, *p*‐value = 0.4935). There are seven variants that should result in a truncated protein, one stop gain in the USP45 gene and six frameshift mutations. Only one sample harboring one of these mutations has a severe SRS *T*‐score of 77. All these protein‐truncating mutations are also observed in the ExAC cohort.

We next tested for associations of each of the 381 common variants (MAF >0.05) in these genes. No SNP was significant after multiple testing corrections (Figure 3b). The top five SNPs are located in Table 2. Since each of these genes has been associated with ASD, we hypothesized that rare and common variants in each of the genes could contribute to SRS. We performed SKAT‐O on the variants located in the autosomal ASD genes altogether, which also showed that there is little evidence to support variants in these 68 ASD genes have a strong effect on SRS *T*‐scores, *p* = 0.431.

While it would be underpowered for any but the largest effect sizes (Figure 2a), for thoroughness we did an unbiased scan of the whole exome. We also examined the polygenic contribution of common variants to the SRS. The common variant analysis was performed on 66,620 variants (Figure 3c). The most nominally significant single SNP is rs527221 located in the *DMPK* gene, which is responsible for causing type 1 myotonic dystrophy (Brook et al., 1992) (Table 2). While there is suggestive evidence for single variants such as rs527221, we calculated the polygenic risk scores (PRS) for each of the individuals in our sample to test if exome wide there are many SNPs of small effect that contribute to the social phenotype in WS. We used the summary statistics from the most recent PGC GWAS on autism spectrum disorders to calculate the PRS for our sample (The Autism Spectrum Disorders Working Group of the Psychiatric Genomics Consortium 2017). We reasoned the polygenic risk of autism would be correlated with the SRS because this is a questionnaire used to assess behaviors that are affected by autism. Variants from the PGC GWAS were included if the *p*‐value for the variant was under the threshold determined by the high‐resolution screen in the PRSice software (Euesden et al., 2015). Interestingly, only the PRS for the motivation subscore was nominally significant (*p* = 0.033), but after permutation to determine an empirical *p*‐value it was not significant (*p* = 0.308). The correlations of the PRS for each of the samples and the subscore as well as total SRS are shown in Supporting Information Fig. S1. Counterintuitively, there is a negative correlation between the PRS and motivation subscore. While this is the largest correlation between the PGS and subscores it implies that more genetic risk for autism leads to a lower and less impaired social motivation *T*‐score. However, given the small sample size and small number of SNPs available from whole‐exome sequencing compared to whole‐genome genotyping we are wary of making strong conclusions from this analysis.

We and others (Klein‐Tasman et al., 2010) have shown that individual subscores of the SRS are affected differently by the deletion of the WSCR. Therefore, we wanted to rule out the possibility that variants are indeed affecting specific subscales of social behavior, but that testing the total SRS score is masking those effects. Thus, in an exploratory manner, we repeated the quantitative trait loci associations for each of the subscores of the SRS using the variants in the WSCR, 71 ASD genes, and the remaining whole exome variants. Since the sample size is small we conducted these associations for exploratory and hypothesis generating purposes. The top five SNPs from each association are reported in Supporting Information Tables [Link], [Link], [Link]. For each of the analyses we see similar variants showing the highest association as were associated with the total SRS, likely due to the high correlation between the SRS and the subscores (Supporting Information Fig. S2). Thus, an analysis of the total SRS was not masking independent genetic effects on each subscale.

## 4 DISCUSSION

Phenotypic variability has been appreciated in many of the symptom domains of WS including the cardiovascular phenotypes, the unique cognitive profile, and in social behavior (Anney et al., 2012; Joyce, Zorich, Pike, Barber, & Dennis, 1996; Porter & Coltheart, 2005). Here, we have described the variability of reciprocal social behavior in a sample of 85 individuals with the typical WS deletion using the SRS‐2. Our results replicate the findings of Klein‐Tasman et al., 2010, revealing that overall individuals with WS have SRS scores that are shifted to the more socially impaired end of the distribution, with most problems relating to the social cognition and restricted and repetitive behavior subscales of the SRS while social motivation is spared.

We also note that sex differences in the general population have been reported previously in the literature for SRS. These sex differences were not consistent with different genetic factors contributing to the SRS in boy and girls, but due to discrepant effects of common genetic and environmental factors on SRS, such as differences in sensitivity to environmental factors or the X‐inactivation phenomenon (Constantino & Todd, 2003). However, we do not see evidence of sex effects in our sample of individuals with WS. The magnitude of the difference between males and females in our sample is similar to what was reported in the general population, so our lack of a significant finding could be due to our small sample size. The standard deviation of the SRS is large in both the general population and still larger in the WS population, so it may also be that larger sample sizes are needed to overcome the considerable variance in the data. The fact that the WS population has a larger standard deviation could also suggest that individuals with the deletion are sensitized to other factors that contribute to variation in the SRS such as background genetic variation or environmental factors.

We performed whole‐exome sequencing on our sample of 85 individuals to test for additional genetic contributions to the variability seen in social behavior in individuals with WS. We used the identified variants to test the hypothesis that genetic variation in the remaining WSCR allele can explain some of the variability in SRS *T*‐scores. Genes in this region have a dosage sensitive effect on social behavior evidenced from the contrasting social phenotypes of the WS deletion and the reciprocal duplication, suggesting that variants in the remaining WSCR allele that affect expression or function of the genes could further contribute to the social phenotype (Merla et al., 2010). We called 120 variants in the WSCR with 55 variants being exonic. We used evidence such as the amino acid change, clinical significance suggested by the ClinVar database, and multiple algorithms to predict the consequences of the variants. Within the WSCR we do not find any variants that cause protein truncation. None of the missense variants are predicted to be deleterious based on the MetaLR composite score. Of the nine variants that have been submitted to ClinVar, all were described as benign or likely benign. A quantitative trait association analysis using the common variants in the region resulted in no SNP that survived multiple testing corrections. The most significant SNP, rs2074754, is a synonymous SNP in the *BAZ1B* gene. This gene encodes for a protein product in the bromodomain protein family that modifies chromatin to affect transcription and has been implicated in the cognitive phenotypes in WS. Knocking down this gene in human derived induced pluripotent stem cells upregulates genes involved in mitosis as well as downregulating genes that are involved in the development of the nervous system (Lalli et al., 2016) The second most nominally significant SNP, rs61438591, is an intronic variant in *GTF2IRD1*, which encodes for a transcription factor that has been suggested to contribute to the cognitive and social behavior deficits (Dai et al., 2009; Howard et al., 2012; Schneider et al., 2012; Tassabehji et al., 2005; Young et al., 2008). If future studies with increased power replicate this association, it would suggest that noncoding variation, perhaps controlling the expression of this gene, might contribute to variation is social behavior. We also tested the association of all variants in the WSCR using SKAT‐O. This test indicated no variants with sufficient effect size were detected in the WSCR.

While we have not shown evidence that variants in the remaining WSCR contribute to the social phenotype in WS, we cannot conclusively discard this hypothesis. However, our study does clearly indicate that the alleles genotyped here are either not causative or exert too small an effect size on SRS for our current power (Figure 2), but it does not rule out variants of small effect on social behavior in the region. Research on other copy number variants associated with ASDs showed that larger CNVs tended to have genes of smaller individual effect size and suggests the phenotype of the overall CNV is due to the cumulative effect of each of those genes (Sanders et al., 2015). Further we did not detect any variants in the gene *GTF2I*, which has been highly suspected of contributing to the social behaviors in WS (Borralleras, Sahun, Pérez‐Jurado, & Campuzano, 2015; Crespi & Hurd, 2014; Dai et al., 2009; Morris et al., 2003; Sakurai et al., 2011). The lack of variant calls in our sample could be due to the fact that *GTF2I* is under stringent purifying selection. Indeed, looking at the ExAC data covering this gene, they show that there are fewer missense variants than expected by chance. ExAC discovered 62 synonymous and 56 missense mutations in 60,706 people (Lek et al., 2016). In our sample of 85 individuals we would expect to see variants in ExAC that have an allele frequency of >0.0059, which is an allele count of one in our sample. There are ten variants with an allele frequency >0.0059 detected in ExAC, only three of which are exonic. Thus, we would need a much larger sample size to investigate coding variants in *GTF2I*. The two linked variants in *GTF2I* that have previously been associated with oxytocin responsiveness and amygdala reactivity, rs1322743 and rs4717907, are intronic and were not covered in our sequencing (Procyshyn et al., 2017; Swartz et al., 2017).

We further used the genetic data to investigate the role of variation in 71 genes that have been associated with ASD. WS and ASD do show phenotypic overlap (Crespi & Procyshyn, 2017; Klein‐Tasman et al., 2009), and we reasoned that these genes should be enriched for functional roles in social behaviors. Likewise, the presence of outlier scores on the SRS that indicated severe impairment, suggested there could be possible second deleterious hits on top of the WS deletion in our dataset. Second hits are expected to be rare but have been observed in WS to explain a case of a child with comorbid seizures (Popp et al., 2016). Inspecting the 1,367 variants discovered in the ASD genes, 313 variants have been previously submitted to ClinVar, none of which show evidence for any pathogenicity. We observed seven protein‐truncating mutations that do not associate with severe SRS *T*‐scores. Several missense mutations were predicted to be deleterious, but there was no association between individuals that had a putative deleterious variant and a more impaired SRS score. Testing the common and rare variants in these genes showed no associations with the social phenotype. Similar results were found when we performed the association analyses on all the variants discovered in the cohort. The most significant SNP was rs527221, a nonsynonymous variant in the *DMPK* gene, which is responsible for causing type 1 myotonic dystrophy, severe childhood forms of which have been associated with ASD (Ekström, Hakenäs‐Plate, Samuelsson, Tulinius, & Wentz, 2008). We also tested if polygenic risk for increased ASD liability is associated with the SRS *T*‐score and subscores. This boosts our ability to detect the impact of many loci with small effects. The largest correlation was between the PRS and the social motivation subscore, although this was not significant.

WS seems to affect specific domains of social behavior as evidenced by significant differences between the subscores of the SRS. This observation led us to an exploratory examination of associations with the subscores of the SRS and test if different genetic variants contribute to each subscore. Overall using variants from the WSCR, ASD genes, or the whole exome identified the same variants as nominally significant. The SRS and the subscores are very correlated, but the social motivation in the WS sample is the least correlated with all other scores. This reflects that fact that social motivation tends to be rated within the normal range in WS, while the other scores are often higher. Interestingly, the whole exome association on the motivation T score leads to the lowest FDR values compared to the other scores, suggesting that there may be more genetic signal when using this subscale. Indeed, this decoupling of the social motivation subscale from other SRS items highlights the possibility that the social motivation subscale might provide useful clinical information going forward; individuals carrying the WSCR deletion yet not showing a spared social motivation might warrant a deeper examination for additional factors impacting their presentation.

There are several limitations to our study that should be addressed in future research. First this study genotyped and assessed only the probands and not their parents. Having genetic information from trios would allow us to distinguish between variants that are inherited or de novo, which would aid in interpretation and prioritization of variants. Furthermore, being able to compare the SRS score of the individual with WS to biparental SRS mean would let us control for effects of background genetic variation (Moreno‐De‐Luca et al., 2015). Second, we are limited to investigating exonic variation. While interpretation of exonic variants is more straightforward because they potentially disrupt coding sequences, and can aid in the detection of deleterious rare variants, we could be missing important regulatory information that is located in promoters or introns of genes. Third, we were not able to control for intellectual functioning of the individuals with WS. The SRS has been reported to not correlate with intellectual functioning (Constantino et al., 2003), but Klein‐Tasman et al., 2010 found significant negative correlations between intellectual functioning and the total SRS *T*‐score when parents completed the report, but not when teachers completed the report. SRS values have been shown to be dependent on levels of expressive language, nonverbal IQ, and behavioral problems. A subset of SRS questions was selected to ameliorate these dependences (Sturm, Kuhfeld, Kasari, & McCracken, 2017). The short form of the SRS as well as other questionnaires that assess adaptive skills and social behaviors could be used in the future to provide supporting information about the social phenotype and underlying genetics in WS. Finally, while our study represents the largest single collection of WS samples reported to date, it is only powered to detect strong effects of common variants due to our small sample size. This is challenging to overcome due to the low prevalence of WS.

In conclusion, we have tested the hypothesis that variation in the remaining WSCR allele affects the social phenotype of individuals with WS, by applying whole exome sequencing to a sample of 85 individuals with typical WS deletions. We show that common and rare variants in the region do not associate with SRS *T*‐scores in our sample. Furthermore, we show that variation outside of the region does not account for the social variability. This is not to say that genetic variation does not play a significant role in phenotypic variability in WS, but that it will require larger sample size to detect. In the future, applying whole genome sequencing to a sample of individuals with WS might elucidate the roles of genetic variation in the regulatory elements. Whole genome data could also allow for more accurate breakpoint determination. Redundant sequences in the low copy number repeat areas at either end of the WS deletion prevent accurate end point detection by CMA. This will be an interesting avenue to pursue in order to investigate how deletion size variation among individuals with typical 1.5 to 1.8 MB deletions contributes to social behavior. For example, Porter et al. showed that those with larger (1.8 Mb deletions) had decreased executive functions (Porter et al., 2012). It is also worth noting that the current genetic data set has additional clinical data available, which can be queried in the future for the presence of more substantial associations with other WS‐related phenotypes.

## References

[mgg3429-bib-0001] Anney, R. , Klei, L. , Pinto, D. , Almeida, J. , Bacchelli, E. , Baird, G. , … Devlin, B. (2012). Individual common variants exert weak effects on the risk for autism spectrum disorders. Human Molecular Genetics, 21, 4781–4792. 10.1093/hmg/dds301 22843504 PMC3471395

[mgg3429-bib-0002] Ashe, A. , Morgan, D. K. , Whitelaw, N. C. , Bruxner, T. J. , Vickaryous, N. K. , Cox, L. L. , … Whitelaw, S. J. (2008). A genome‐wide screen for modifiers of transgene variegation identifies genes with critical roles in development. Genome Biology, 9, R182 10.1186/gb-2008-9-12-r182 19099580 PMC2646286

[mgg3429-bib-0003] Borralleras, C. , Sahun, I. , Pérez‐Jurado, L. A. , & Campuzano, V. (2015). Intracisternal Gtf2i gene therapy ameliorates deficits in cognition and synaptic plasticity of a mouse model of Williams–Beuren syndrome. Molecular Therapy, 23, 1691–1699. 10.1038/mt.2015.130 26216516 PMC4817950

[mgg3429-bib-0004] Brawn, G. , & Porter, M. (2014). Adaptive functioning in Williams syndrome and its relation to demographic variables and family environment. Research in Developmental Disabilities, 35, 3606–3623. 10.1016/j.ridd.2014.08.012 25310713

[mgg3429-bib-0005] Brook, J. D. , McCurrach, M. E. , Harley, H. G. , Buckler, A. J. , Church, D. , Aburatani, H. , … Housman, D. E. (1992). Molecular basis of myotonic dystrophy: Expansion of a trinucleotide (CTG) repeat at the 3′ end of a transcript encoding a protein kinase family member. Cell, 68, 799–808. 10.1016/0092-8674(92)90154-5 1310900

[mgg3429-bib-0006] Constantino, J. N. , Davis, S. A. , Todd, R. D. , Schindler, M. K. , Gross, M. M. , Brophy, S. L. , … Reich, W. (2003). Validation of a brief quantitative measure of autistic traits: Comparison of the social responsiveness scale with the autism diagnostic interview‐revised. Journal of Autism and Developmental Disorders, 33, 427–433. 10.1023/A:1025014929212 12959421

[mgg3429-bib-0007] Constantino, J. N. , & Todd, R. D. (2003). Autistic traits in the general population: A twin study. Archives of General Psychiatry, 60, 524–530. 10.1001/archpsyc.60.5.524 12742874

[mgg3429-bib-0008] Crespi, B. J. , & Hurd, P. L. (2014). Cognitive‐behavioral phenotypes of Williams's syndrome are associated with genetic variation in the GTF2I gene, in a healthy population. BMC Neuroscience, 15, 127 10.1186/s12868-014-0127-1 25429715 PMC4247780

[mgg3429-bib-0009] Crespi, B. J. , & Procyshyn, T. L. (2017). Williams syndrome deletions and duplications: Genetic windows to understanding anxiety, sociality, autism, and schizophrenia. Neuroscience and Biobehavioral Reviews, 79, 14–26. 10.1016/j.neubiorev.2017.05.004 28499504

[mgg3429-bib-0010] Dai, L. , Bellugi, U. , Chen, X.‐N. , Pulst‐Korenberg, A. M. , Järvinen‐Pasley, A. , Tirosh‐Wagner, T. , Eis, P. S. , … Koenberg, J. R. (2009). Is it Williams syndrome? GTF2IRD1 implicated in visual–spatial construction and GTF2I in sociability revealed by high resolution arrays American Journal of Medical Genetics Part A, 149A, 302–314. 10.1002/ajmg.a.32652 19205026 PMC2650741

[mgg3429-bib-0011] Danecek, P. , Auton, A. , Abecasis, G. , Albers, C. A. , Banks, E. , DePristo, M. A. , … 1000 Genomes Project Analysis Group . (2011). The variant call format and VCFtools. Bioinformatics, 27, 2156–2158. 10.1093/bioinformatics/btr330 21653522 PMC3137218

[mgg3429-bib-0012] Delio, M. , Pope, K. , Wang, T. , Samanich, J. , Haldeman‐Englert, C. R. , Kaplan, P. , … Babcock, M. (2013). Spectrum of elastin sequence variants and cardiovascular phenotypes in 49 patients with Williams‐Beuren syndrome. American Journal of Medical Genetics. Part A, 161, 527–533. 10.1002/ajmg.a.35784 23401415

[mgg3429-bib-0013] DePristo, M. A. , Banks, E. , Poplin, R. , Garimella, K. V. , Maguire, J. R. , Hartl, C. , … Daly, M. J. (2011). A framework for variation discovery and genotyping using next‐generation DNA sequencing data. Nature Genetics, 43, 491–498. 10.1038/ng.806 21478889 PMC3083463

[mgg3429-bib-0014] Dong, C. , Wei, P. , Jian, X. , Gibbs, R. , Boerwinkle, E. , Wang, K. , & Liu, X. (2015). Comparison and integration of deleteriousness prediction methods for nonsynonymous SNVs in whole exome sequencing studies. Human Molecular Genetics, 24, 2125–2137. 10.1093/hmg/ddu733 25552646 PMC4375422

[mgg3429-bib-0015] Doyle, T. F. , Bellugi, U. , Korenberg, J. R. , & Graham, J. (2004). “Everybody in the world is my friend” hypersociability in young children with Williams syndrome. American Journal of Medical Genetics. Part A, 124A, 263–273. 10.1002/(ISSN)1096-8628 14708099

[mgg3429-bib-0016] Dykens, E. M. (2003). Anxiety, fears, and phobias in persons with Williams syndrome. Developmental Neuropsychology, 23, 291–316. 10.1207/S15326942DN231&2_13 12730029

[mgg3429-bib-0017] Einfeld, S. L. , Tonge, B. J. , & Florio, T. (1997). Behavioral and emotional disturbance in individuals with Williams syndrome. American Journal of Mental Retardation, 102, 45–53. 10.1352/0895-8017(1997)102lt;0045:BAEDIIgt;2.0.CO;2 9241407

[mgg3429-bib-0018] Einfeld, S. L. , Tonge, B. J. , & Rees, V. W. (2001). Longitudinal course of behavioral and emotional problems in Williams syndrome. American Journal of Mental Retardation, 106, 73–81. 10.1352/0895-8017(2001)106lt;0073:LCOBAEgt;2.0.CO;2 11246715

[mgg3429-bib-0019] Ekström, A.‐B. , Hakenäs‐Plate, L. , Samuelsson, L. , Tulinius, M. , & Wentz, E. (2008). Autism spectrum conditions in myotonic dystrophy type 1: A study on 57 individuals with congenital and childhood forms. American Journal of Medical Genetics. Part B, Neuropsychiatric Genetics: The Official Publication of the International Society of Psychiatric Genetics., 147B, 918–926. 10.1002/ajmg.b.30698 18228241

[mgg3429-bib-0020] Euesden, J. , Lewis, C. M. , & O'Reilly, P. F. (2015). PRSice: Polygenic risk score software. Bioinformatics, 31, 1466–1468. 10.1093/bioinformatics/btu848 25550326 PMC4410663

[mgg3429-bib-0021] Ewart, A. K. , Morris, C. A. , Ensing, G. J. , Loker, J. , Moore, C. , Leppert, M. , & Keating, M. (1993). A human vascular disorder, supravalvular aortic stenosis, maps to chromosome 7. Proceedings of the National Academy of Sciences, 90, 3226–3230. 10.1073/pnas.90.8.3226 PMC46272 8475063

[mgg3429-bib-0022] Fujiwara, T. , Sanada, M. , Kofuji, T. , & Akagawa, K. (2016). Unusual social behavior in HPC‐1/syntaxin1A knockout mice is caused by disruption of the oxytocinergic neural system. Journal of Neurochemistry, 138, 117–123. 10.1111/jnc.13634 27059771

[mgg3429-bib-0023] Gao, M. C. , Bellugi, U. , Dai, L. , Mills, D. L. , Sobel, E. M. , Lange, K. , & Korenberg, J. R. (2010). Intelligence in Williams syndrome is related to STX1A, which encodes a component of the presynaptic SNARE complex. PLoS ONE, 5, e10292 10.1371/journal.pone.0010292 20422020 PMC2858212

[mgg3429-bib-0024] Gosch, A. , & Pankau, R. (1997). Personality characteristics and behaviour problems in individuals of different ages with Williams syndrome. Developmental Medicine and Child Neurology, 39, 527–533. 9295848 10.1111/j.1469-8749.1997.tb07481.x

[mgg3429-bib-0025] van Hagen, J. M. , van der Geest, J. N. , van der Giessen, R. S. , Lagers‐van Haselen, G. C. , Eussen, H. J. F. M. M. , Gille, J. J. P. , … De Zeeuw, C. I. (2007). Contribution of CYLN2 and GTF2IRD1 to neurological and cognitive symptoms in Williams syndrome. Neurobiology of Diseases, 26, 112–124. 10.1016/j.nbd.2006.12.009 17270452

[mgg3429-bib-0026] Hoogenraad, C. C. , Koekkoek, B. , Akhmanova, A. , Krugers, H. , Dortland, B. , Miedema, M. , … Galjart, N. (2002). Targeted mutation of Cyln2 in the Williams syndrome critical region links CLIP‐115 haploinsufficiency to neurodevelopmental abnormalities in mice. Nature Genetics, 32, 116–127. 10.1038/ng954 12195424

[mgg3429-bib-0027] Howard, M. L. , Palmer, S. J. , Taylor, K. M. , Arthurson, G. J. , Spitzer, M. W. , Du, X. , … Hannan, A. J. (2012). Mutation of Gtf2ird1 from the Williams‐Beuren syndrome critical region results in facial dysplasia, motor dysfunction, and altered vocalisations. Neurobiology of Diseases, 45, 913–922. 10.1016/j.nbd.2011.12.010 22198572

[mgg3429-bib-0028] Jabbi, M. , Chen, Q. , Turner, N. , Kohn, P. , White, M. , Kippenhan, J. S. , … Berman, K. F. (2015). Variation in the Williams syndrome GTF2I gene and anxiety proneness interactively affect prefrontal cortical response to aversive stimuli. Translational Psychiatry, 5, e622 10.1038/tp.2015.98 26285132 PMC4564573

[mgg3429-bib-0029] Järvinen, A. , Korenberg, J. R. , & Bellugi, U. (2013). The social phenotype of Williams syndrome. Current Opinion in Neurobiology, 23, 414–422. 10.1016/j.conb.2012.12.006 23332975 PMC4326252

[mgg3429-bib-0030] Joyce, C. A. , Zorich, B. , Pike, S. J. , Barber, J. C. , & Dennis, N. R. (1996). Williams‐Beuren syndrome: Phenotypic variability and deletions of chromosomes 7, 11, and 22 in a series of 52 patients. Journal of Medical Genetics, 33, 986–992. 10.1136/jmg.33.12.986 9004128 PMC1050807

[mgg3429-bib-0031] Kircher, M. , Witten, D. M. , Jain, P. , O'Roak, B. J. , Cooper, G. M. , & Shendure, J. (2014). A general framework for estimating the relative pathogenicity of human genetic variants. Nature Genetics, 46, 310–315. 10.1038/ng.2892 24487276 PMC3992975

[mgg3429-bib-0032] Klein‐Tasman, B. P. , Li‐Barber, K. T. , & Magargee, E. T. (2010). Honing in on the social phenotype in Williams syndrome using multiple measures and multiple raters. Journal of Autism and Developmental Disorders, 41, 341–351. 10.1007/s10803-010-1060-5 PMC3020248 20614173

[mgg3429-bib-0033] Klein‐Tasman, B. P. , & Mervis, C. B. (2003). Distinctive personality characteristics of 8‐, 9‐, and 10‐year‐olds with Williams syndrome. Developmental Neuropsychology, 23, 269–290. 10.1207/S15326942DN231&2_12 12730028

[mgg3429-bib-0034] Klein‐Tasman, B. P. , & Mervis, C. B. (2018). Autism spectrum symptomatology among children with duplication 7q11.23 syndrome. Journal of Autism and Developmental Disorders, 48, 1–13. 29307037 10.1007/s10803-017-3439-z PMC6003247

[mgg3429-bib-0035] Klein‐Tasman, B. P. , Mervis, C. B. , Lord, C. , & Phillips, K. D. (2007). Socio‐communicative deficits in young children with Williams syndrome: Performance on the autism diagnostic observation schedule. Child Neuropsychology, 13, 444–467. 10.1080/09297040601033680 17805996

[mgg3429-bib-0036] Klein‐Tasman, B. P. , Phillips, K. D. , Lord, C. E. , Mervis, C. B. , & Gallo, F. (2009). Overlap with the Autism spectrum in young children with Williams syndrome. Journal of Developmental and Behavioral Pediatrics, 30, 289–299. 10.1097/DBP.0b013e3181ad1f9a 19668090 PMC2763277

[mgg3429-bib-0037] Lalli, M. A. , Jang, J. , Park, J.‐H. C. , Wang, Y. , Guzman, E. , Zhou, H. , … Kosik, K. S. (2016). Haploinsufficiency of BAZ1B contributes to Williams syndrome through transcriptional dysregulation of neurodevelopmental pathways. Human Molecular Genetics, 25, 1294–1306. 10.1093/hmg/ddw010 26755828

[mgg3429-bib-0038] Landrum, M. J. , Lee, J. M. , Benson, M. , Brown, G. , Chao, C. , Chitipiralla, S. , … Maglott, D. R. (2016). ClinVar: Public archive of interpretations of clinically relevant variants. Nucleic Acids Research, 44, D862–D868. 10.1093/nar/gkv1222 26582918 PMC4702865

[mgg3429-bib-0039] Lee, S. , Wu, M. C. , & Lin, X. (2012). Optimal tests for rare variant effects in sequencing association studies. Biostatistics, 13, 762–775. 10.1093/biostatistics/kxs014 22699862 PMC3440237

[mgg3429-bib-0040] Lek, M. , Karczewski, K. J. , Minikel, E. V. , Samocha, K. E. , Banks, E. , Fennell, T. , … Exome Aggregation Consortium . (2016). Analysis of protein‐coding genetic variation in 60,706 humans. Nature, 536, 285–291. 10.1038/nature19057 27535533 PMC5018207

[mgg3429-bib-0041] Leyfer, O. T. , Woodruff‐Borden, J. , Klein‐Tasman, B. P. , Fricke, J. S. , & Mervis, C. B. (2006). Prevalence of psychiatric disorders in 4–16‐year‐olds with Williams syndrome. American Journal of Medical Genetics, Part B, Neuropsychiatric Genetics, 141B, 615–622. 10.1002/(ISSN)1552-485X PMC2561212 16823805

[mgg3429-bib-0042] Li, D. Y. , Brooke, B. , Davis, E. C. , Mecham, R. P. , Sorensen, L. K. , Boak, B. B. , … Keating, M. T. (1998). Elastin is an essential determinant of arterial morphogenesis. Nature, 393, 276–280. 10.1038/30522 9607766

[mgg3429-bib-0043] Li, H. , & Durbin, R. (2009). Fast and accurate short read alignment with Burrows‐Wheeler transform. Bioinformatics, 25, 1754–1760. 10.1093/bioinformatics/btp324 19451168 PMC2705234

[mgg3429-bib-0044] Li, H. , Handsaker, B. , Wysoker, A. , Fennell, T. , Ruan, J. , Homer, N. , … Durbin, R. (2009). The sequence alignment/map format and SAMtools. Bioinformatics, 25, 2078–2079. 10.1093/bioinformatics/btp352 19505943 PMC2723002

[mgg3429-bib-0045] Li, D. Y. , Toland, A. E. , Boak, B. B. , Atkinson, D. L. , Ensing, G. J. , Morris, C. A. , & Keating, M. T. (1997). Elastin point mutations cause an obstructive vascular disease, supravalvular aortic stenosis. Human Molecular Genetics, 6, 1021–1028. 10.1093/hmg/6.7.1021 9215670

[mgg3429-bib-0046] Liu, X. , Wu, C. , Li, C. , & Boerwinkle, E. (2016). dbNSFP v3.0: A one‐stop database of functional predictions and annotations for human nonsynonymous and splice‐site SNVs. Human Mutation, 37, 235–241. 10.1002/humu.22932 26555599 PMC4752381

[mgg3429-bib-0047] Manolio, T. A. , Collins, F. S. , Cox, N. J. , Goldstein, D. B. , Hindorff, L. A. , Hunter, D. J. , … Visscher, P. M. (2009). Finding the missing heritability of complex diseases. Nature, 461, 747–753. 10.1038/nature08494 19812666 PMC2831613

[mgg3429-bib-0048] Meng, Y. , Zhang, Y. , Tregoubov, V. , Janus, C. , Cruz, L. , Jackson, M. , … Jia, Z. (2002). Abnormal spine morphology and enhanced LTP in LIMK‐1 knockout mice. Neuron, 35, 121–133. 10.1016/S0896-6273(02)00758-4 12123613

[mgg3429-bib-0049] Merla, G. , Brunetti‐Pierri, N. , Micale, L. , & Fusco, C. (2010). Copy number variants at Williams‐Beuren syndrome 7q11.23 region. Human Genetics, 128, 3–26. 10.1007/s00439-010-0827-2 20437059

[mgg3429-bib-0050] Mervis, C. B. , Robinson, B. F. , Bertrand, J. , Morris, C. A. , Klein‐Tasman, B. P. , & Armstrong, S. C. (2000). The Williams syndrome cognitive profile. Brain and Cognition, 44, 604–628. 10.1006/brcg.2000.1232 11104544

[mgg3429-bib-0051] Moreno‐De‐Luca, A. , Evans, D. W. , Boomer, K. B. , Hanson, E. , Bernier, R. , Goin‐Kochel, R. P. , … Ledbetter, D. H. (2015). The role of parental cognitive, behavioral, and motor profiles in clinical variability in individuals with chromosome 16p11.2 deletions. JAMA Psychiatry, 72, 119–126. 10.1001/jamapsychiatry.2014.2147 25493922

[mgg3429-bib-0052] Morris, C. A. , Mervis, C. B. , Hobart, H. H. , Gregg, R. G. , Bertrand, J. , Ensing, G. J. , … Stock, A. D. (2003). GTF2I hemizygosity implicated in mental retardation in Williams syndrome: Genotype–phenotype analysis of five families with deletions in the Williams syndrome region. American Journal of Medical Genetics. Part A, 123A, 45–59. 10.1002/(ISSN)1096-8628 14556246

[mgg3429-bib-0053] Popp, B. , Trollmann, R. , Büttner, C. , Caliebe, A. , Thiel, C. T. , Hüffmeier, U. , … Zweier, C. (2016). Do the exome: A case of Williams‐Beuren syndrome with severe epilepsy due to a truncating de novo variant in GABRA1. European Journal of Medical Genetics, 59, 549–553. 10.1016/j.ejmg.2016.09.002 27613244

[mgg3429-bib-0054] Porter, M. A. , & Coltheart, M. (2005). Cognitive heterogeneity in Williams syndrome. Developmental Neuropsychology, 27, 275–306. 10.1207/s15326942dn2702_5 15753050

[mgg3429-bib-0055] Porter, M. A. , Dobson‐Stone, C. , Kwok, J. B. J. , Schofield, P. R. , Beckett, W. , & Tassabehji, M. (2012). A role for transcription factor GTF2IRD2 in executive function in Williams‐Beuren syndrome. PLoS ONE, 7, e47457 10.1371/journal.pone.0047457 23118870 PMC3485271

[mgg3429-bib-0056] Procyshyn, T. L. , Spence, J. , Read, S. , Watson, N. V. , & Crespi, B. J. (2017). The Williams syndrome prosociality gene GTF2I mediates oxytocin reactivity and social anxiety in a healthy population. Biology Letters, 13, 20170051 10.1098/rsbl.2017.0051 28424317 PMC5414696

[mgg3429-bib-0057] Purcell, S. , Cherny, S. S. , & Sham, P. C. (2003). Genetic Power Calculator: Design of linkage and association genetic mapping studies of complex traits. Bioinformatics, 19, 149–150. 10.1093/bioinformatics/19.1.149 12499305

[mgg3429-bib-0058] Purcell, S. , Neale, B. , Todd‐Brown, K. , Thomas, L. , Ferreira, M. A. R. , Bender, D. , … Sham, P. C. (2007). PLINK: A tool set for whole‐genome association and population‐based linkage analyses. American Journal of Human Genetics, 81, 559–575. 10.1086/519795 17701901 PMC1950838

[mgg3429-bib-0059] Reilly, J. , Klima, E. S. , & Bellugi, U. (1990). Once more with feeling: Affect and language in atypical populations. Development and Psychopathology, 2, 367–391. 10.1017/S0954579400005782

[mgg3429-bib-0060] Richards, C. , Jones, C. , Groves, L. , Moss, J. , & Oliver, C. (2015). Prevalence of autism spectrum disorder phenomenology in genetic disorders: A systematic review and meta‐analysis. Lancet Psychiatry, 2, 909–916. 10.1016/S2215-0366(15)00376-4 26341300

[mgg3429-bib-0061] Sakurai, T. , Dorr, N. P. , Takahashi, N. , McInnes, L. A. , Elder, G. A. , & Buxbaum, J. D. (2011). Haploinsufficiency of Gtf2i, a gene deleted in Williams Syndrome, leads to increases in social interactions. Autism Research, 4, 28–39. 10.1002/aur.169 21328569

[mgg3429-bib-0062] Sanders, S. J. , He, X. , Willsey, A. J. , Ercan‐Sencicek, A. G. , Samocha, K. E. , Cicek, A. E. , … State, M. W. (2015). Insights into autism spectrum disorder genomic architecture and biology from 71 risk loci. Neuron, 87, 1215–1233. 10.1016/j.neuron.2015.09.016 26402605 PMC4624267

[mgg3429-bib-0063] Schneider, T. , Skitt, Z. , Liu, Y. , Deacon, R. M. J. , Flint, J. , Karmiloff‐Smith, A. , … Tassabehji, M. (2012). Anxious, hypoactive phenotype combined with motor deficits in Gtf2ird1 null mouse model relevant to Williams syndrome. Behavioral Brain Research, 233, 458–473. 10.1016/j.bbr.2012.05.014 22652393

[mgg3429-bib-0064] Sturm, A. , Kuhfeld, M. , Kasari, C. , & McCracken, J. T. (2017). Development and validation of an item response theory‐based Social Responsiveness Scale short form. Journal of Child Psychology and Psychiatry, 58, 1053–1061. 10.1111/jcpp.12731 28464350

[mgg3429-bib-0065] Swartz, J. R. , Waller, R. , Bogdan, R. , Knodt, A. R. , Sabhlok, A. , Hyde, L. W. , & Hariri, A. R. (2017). A common polymorphism in a Williams syndrome gene predicts amygdala reactivity and extraversion in healthy adults. Biological Psychiatry, 81, 203–210. 10.1016/j.biopsych.2015.12.007 26853120 PMC4909599

[mgg3429-bib-0066] Switaj, D. M. (2001). Identification and measurement of anxiety and obsessive‐compulsive tendencies in the Williams Syndrome behavioral phenotype.

[mgg3429-bib-0067] Tager‐Flusberg, H. , & Sullivan, K. (2000). A componential view of theory of mind: Evidence from Williams syndrome. Cognition, 76, 59–90. 10.1016/S0010-0277(00)00069-X 10822043

[mgg3429-bib-0068] Tassabehji, M. , Hammond, P. , Karmiloff‐Smith, A. , Thompson, P. , Thorgeirsson, S. S. , Durkin, M. E. , … Donnai, D. (2005). GTF2IRD1 in craniofacial development of humans and mice. Science, 310, 1184–1187. 10.1126/science.1116142 16293761

[mgg3429-bib-0069] The Autism Spectrum Disorders Working Group of the Psychiatric Genomics Consortium (2017). Meta‐analysis of GWAS of over 16,000 individuals with autism spectrum disorder highlights a novel locus at 10q24.32 and a significant overlap with schizophrenia. Molecular Autism, 8, 1–17 10.1186/s13229-017-0137-9. 28540026 PMC5441062

[mgg3429-bib-0070] Turner, S. D. (2014). qqman: an R package for visualizing GWAS results using Q‐Q and manhattan plots. BioRxiv 005165.

[mgg3429-bib-0071] Wang, K. , Li, M. , & Hakonarson, H. (2010). ANNOVAR: Functional annotation of genetic variants from high‐throughput sequencing data. Nucleic Acids Research, 38, e164 10.1093/nar/gkq603 20601685 PMC2938201

[mgg3429-bib-0072] Williams, J. C. P. , Barratt‐Boyes, B. G. , & Lowe, J. B. (1961). Supravalvular aortic stenosis. Circulation, 24, 1311–1318. 10.1161/01.CIR.24.6.1311 14007182

[mgg3429-bib-0073] Young, E. J. , Lipina, T. , Tam, E. , Mandel, A. , Clapcote, S. J. , Bechard, A. R. , … Osborne, L. R. (2008). Reduced fear and aggression and altered serotonin metabolism in Gtf2ird1‐targeted mice. Genes, Brain, and Behavior, 7, 224–234. 10.1111/j.1601-183X.2007.00343.x PMC2883608 17680805

