# Supplementary material for: Exome sequencing of 85 Williams–Beuren syndrome cases rules out coding variation as a major contributor to remaining variance in social behavior
Source: Mol Genet Genomic Med. 2018 Jul 15;6(5):749–65. doi: 10.1002/mgg3.429 (PMC6160704; doi:10.1002/mgg3.429)
Supplement: Supplementary file 1 [file MGG3-6-749-s001.pdf]

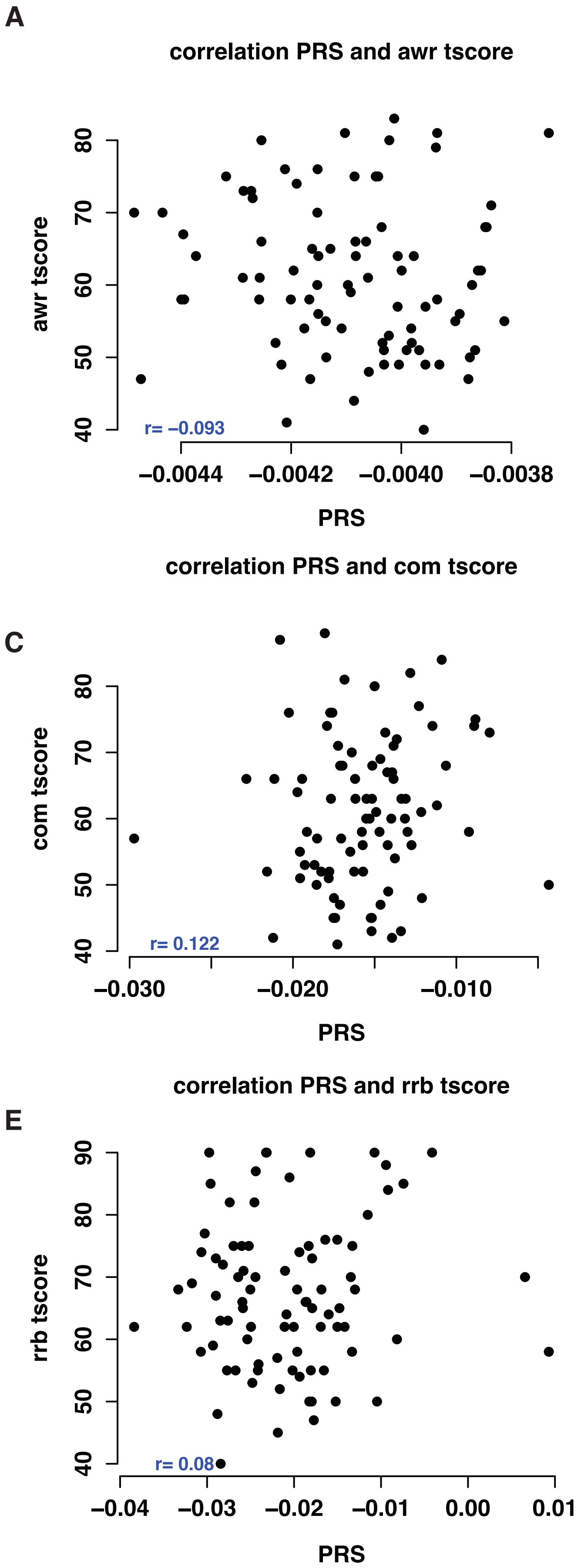


**Supplemental Figure 1:Polygenic Risk Score correlation with SRS and SRS subscores. A-F** Panels show the correlation between the polygenic risk score (PRS) for the sub score of the SRS calculated using variatns from the PGC ASD GWAS that fall below the p-value threshold calculated from the best-fit PRS. Pearson correlation values between the samples PRS and the SRS subscore shown as the inset.
