# Supplementary material for: Exome sequencing of 85 Williams–Beuren syndrome cases rules out coding variation as a major contributor to remaining variance in social behavior
Source: Mol Genet Genomic Med. 2018 Jul 15;6(5):749–65. doi: 10.1002/mgg3.429 (PMC6160704; doi:10.1002/mgg3.429)
Supplement: Supplementary file 2 [file MGG3-6-749-s002.pdf]

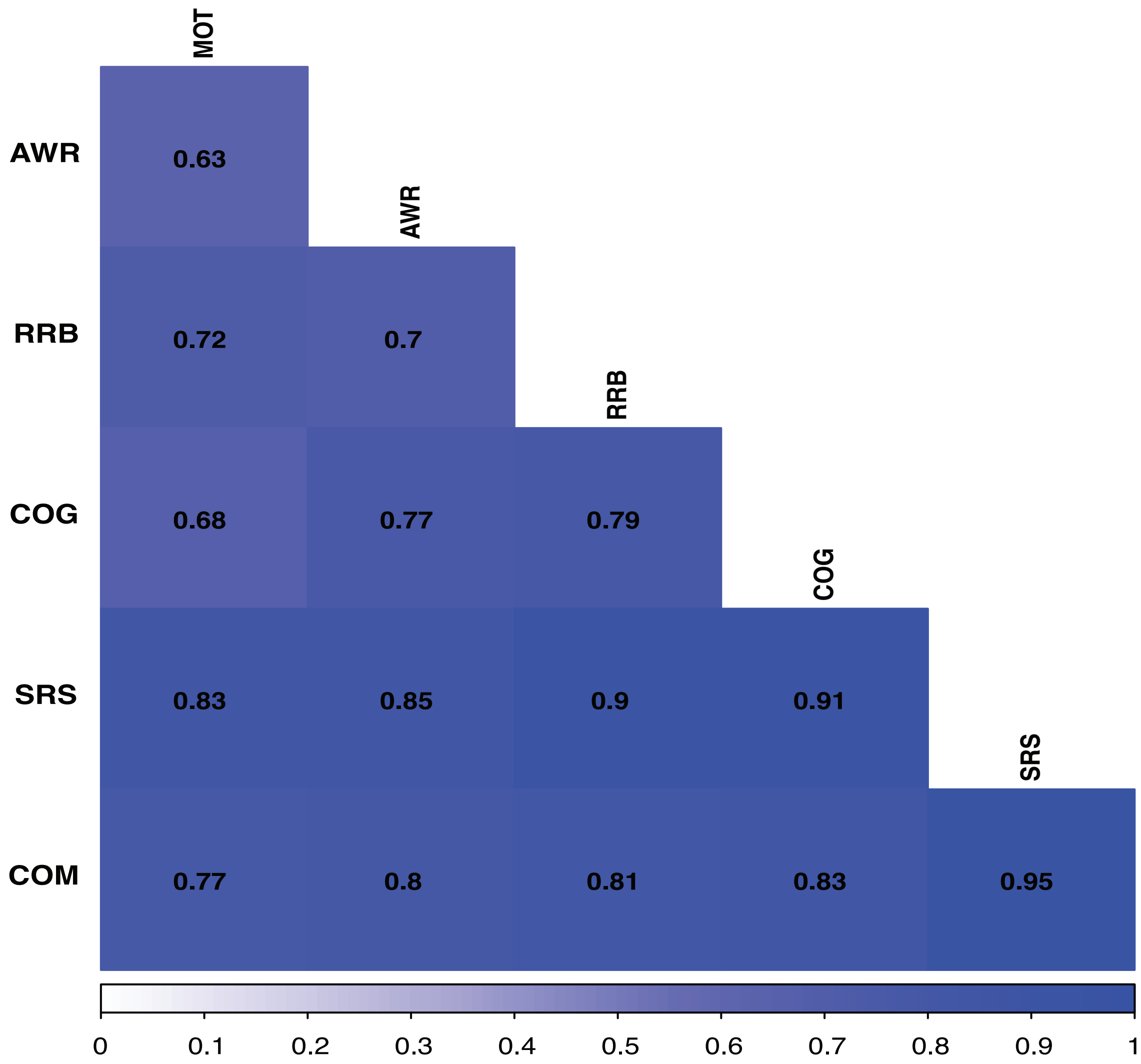

Supplemental Figure 2: SRS and sub scales are correlated. Heatmap display of the Pearson correlation values of the SRS and sub scale T-scores in 85 individuals with WS. Values of the correlation are labeled in the plot.
