# Supplementary material for: Exome sequencing of 85 Williams–Beuren syndrome cases rules out coding variation as a major contributor to remaining variance in social behavior
Source: Mol Genet Genomic Med. 2018 Jul 15;6(5):749–65. doi: 10.1002/mgg3.429 (PMC6160704; doi:10.1002/mgg3.429)
Supplement: Supplementary file 5 [file MGG3-6-749-s005.docx]

| SNP | Alt allele | MAF | Transcript^a^ | Gene | Consequence | Beta | 95% Confidence interval | Raw  p-value | FDR | SRS sub category |
| --- | --- | --- | --- | --- | --- | --- | --- | --- | --- | --- |
| rs3812316 | G | 0.10 | NM_032951 | *MLXIPL* | p.Q241H | 4.817 | 1.146-8.487 | 0.01206 | .4101 | AWR |
| rs13235543 | T | 0.12 | NM_032954 | *MLXIPL* | p.P342P | 3.399 | 0.05103-6.746 | 0.05016 | .5083 | AWR |
| rs2074754 | T | 0.4 | NM_032408 | *BAZ1B* | p.S679S | 2.24 | -0.5935 – 4.54 | 0.06004 | .5083 | AWR |
| rs61438591 | C | 0.2 | . | *GTF2IRD1* | intronic | 2.426 | -0.3677 – 5.22 | 0.09284 | .5083 | AWR |
| rs2071307 | A | 0.47 | NM_001081752 | *ELN* | p.G412S | 1.963 | -0.2993 – 4.225 | 0.09305 | .5083 | AWR |
| rs2074754 | T | 0.4 | NM_032408 | *BAZ1B* | p.S679S | 4.145 | 1.496-6.794 | 0.003006 | 0.1022 | COG |
| rs61438591 | C | 0.2 | . | *GTF2IRD1* | intronic | 3.578 | 0.2897-6.867 | 0.03618 | 0.6151 | COG |
| rs17851629 | G | 0.21 | NM_016328 | *GTF2IRD1* | E171E | 3.129 | -0.1129 – 6.37 | 0.06229 | 0.706 | COG |
| rs61010704 | G | 0.23 | . | *MLXIPL* | intronic | 2.377 | -0.872 – 5.626 | 0.1559 | 0.8179 | COG |
| rs7795181 | C | 0.22 | . | *VPS37D* | intronic | -2.082 | -5.346 – 1.183 | 0.2153 | 0.8179 | COG |
| rs2074754 | T | 0.4 | NM_032408 | *BAZ1B* | p.S679S | 3.172 | 0.7265 – 5.617 | 0.01307 | 0.2675 | COM |
| rs61438591 | C | 0.2 | . | *GTF2IRD1* | intronic | 3.732 | 0.7711 – 6.692 | 0.01573 | 0.2675 | COM |
| rs17851629 | G | 0.21 | NM_016328 | *GTF2IRD1* | E171E | 3.241 | 0.2964 – 6.186 | 0.0341 | 0.3865 | COM |
| rs3812316 | G | 0.10 | NM_032951 | *MLXIPL* | p.Q241H | 3.122 | -0.995 – 7.238 | 0.1414 | 0.7693 | COM |
| rs3135698 | C | 0.06 | . | *RFC2* | intronic | -4.712 | -11.52 – 2.093 | 0.1787 | 0.7693 | COM |
| rs2074754 | T | 0.4 | NM_032408 | *BAZ1B* | p.S679S | 2.411 | 0.301 – 4.521 | 0.02808 | 0.8893 | MOT |
| rs61438591 | C | 0.2 | . | *GTF2IRD1* | intronic | 2.441 | -.1236 – 5.007 | 0.06597 | 0.8893 | MOT |
| rs17851629 | G | 0.21 | NM_016328 | *GTF2IRD1* | E171E | 1.862 | -0.6716 – 4.396 | 0.1538 | 0.8893 | MOT |
| rs76029572 | G | 0.07 | NM_012453 | *TBL2* | p.E8Q | -2.966 | -7.008 – 1.075 | 0.1543 | 0.8893 | MOT |
| rs2240357 | C | 0.23 | NM_016328 | *GTF2IRD1* | p.Y404Y | 1.745 | -0.7424 – 4.232 | 0.1731 | 0.8893 | MOT |
| rs2074754 | T | 0.4 | NM_032408 | *BAZ1B* | p.S679S | 2.436 | -0.2729 – 5.146 | 0.08205 | 0.7104 | RRB |
| rs61438591 | C | 0.2 | . | *GTF2IRD1* | intronic | 2.762 | -0.4886 – 6.012 | 0.09996 | 0.7104 | RRB |
| rs2071307 | A | 0.47 | NM_001081752 | *ELN* | p.G412S | 2.222 | -0.4162 – 4.86 | 0.1029 | 0.7104 | RRB |
| rs61010704 | G | 0.25 | . | *MLXIPL* | intronic | 2.476 | -0.6671 – 5.619 | 0.1269 | 0.7104 | RRB |
| rs3812316 | G | 0.10 | NM_032951 | *MLXIPL* | p.Q241H | 3.245 | -1.159 – 7.649 | 0.1528 | 0.7104 | RRB |
| ^a^ “.” Refers to information that is not applicable | | | | | | | | | | |
