# Supplementary material for: Exome sequencing of 85 Williams–Beuren syndrome cases rules out coding variation as a major contributor to remaining variance in social behavior
Source: Mol Genet Genomic Med. 2018 Jul 15;6(5):749–65. doi: 10.1002/mgg3.429 (PMC6160704; doi:10.1002/mgg3.429)
Supplement: Supplementary file 6 [file MGG3-6-749-s006.docx]

| SNP | Alt allele | MAF | Transcript^a^ | Gene | Consequence | Beta | 95% Confidence interval | Raw  p-value | FDR | SRS sub category |
| --- | --- | --- | --- | --- | --- | --- | --- | --- | --- | --- |
| rs45599933 | A | 0.06 | . | *CAPN12* | intronic | 11.72 | 4.596 – 18.85 | 0.001854 | 0.1735 | AWR |
| rs12983010 | G | 0.07 | NM_144691 | *CAPN12* | p.C287R | 10.47 | 4.028 – 16.91 | 0.002085 | 0.1735 | AWR |
| rs3733615 | G | .16 | NM_001148 | *ANK2* | p.Q2370Q | 6.806 | 2.486 – 11.13 | 0.0028 | 0.1735 | AWR |
| rs33966911 | T | 0.11 | NM_001148 | *ANK2* | p.P1823P | 7.609 | 2.764 – 12.45 | 0.002885 | 0.1735 | AWR |
| rs28377576 | C | 0.11 | NM_001148 | *ANK2* | p.V2369A | 7.578 | 2.734 – 12.45 | 0.002991 | 0.1735 | AWR |
| rs3750354 | T | 0.39 | . | *PHF2* | intronic | -6.727 | -10.58 – -2.875 | 0.0009956 | 0.1897 | COG |
| rs7036592 | T | 0.39 | . | *PHF2* | intronic | -6.727 | -10.58 – -2.875 | 0.0009956 | 0.1897 | COG |
| rs10992813 | A | 0.37 | . | *PHF2* | intronic | -6.476 | -10.43 – -2.527 | 0.001922 | 0.2441 | COG |
| rs3763605 | G | .63 | . | *PHF2* | intronic | 5.41 | 1.399 – 9.421 | 0.009964 | 0.5607 | COG |
| rs3750358 | C | .63 | . | *PHF2* | intronic | 5.311 | 1.276 – 9.345 | 0.01178 | 0.5607 | COG |
| rs112318565 | G | 0.06 | . | *ARID1B* | intronic | 11.63 | 3.582 – 19.67 | 0.005892 | 0.8653 | COM |
| rs12553775 | A | 0.11 | . | *PHF2* | intronic | 7.386 | 1.482 – 13.29 | 0.01647 | 0.8653 | COM |
| rs140682 | C | 0.48 | NM_000810 | *GABRA5* | p.V202V | -4.218 | -7.659 – -0.7778 | 0.01867 | 0.8653 | COM |
| rs4351684 | G | 0.51 | . | *ILF2* | intronic | -4.662 | -8.546 – -0.7782 | 0.02119 | 0.8653 | COM |
| rs1805482 | A | 0.35 | NM_000834 | *GRIN2B* | p.S555S | 4.615 | 0.6839 – 8.546 | 0.0241 | 0.8653 | COM |
| rs30612 | C | 0.84 | NM_007118 | *TRIO* | p.T1700T | 6.392 | 2.773 – 10.01 | 0.0008803 | 0.3354 | MOT |
| rs12983010 | G | 0.07 | NM_144691 | *CAPN12* | p.C287R | 8.842 | 2.839 – 14.84 | 0.005044 | 0.6218 | MOT |
| rs45599933 | A | 0.06 | . | *CAPN12* | intronic | 9.766 | 3.115 – 16.42 | 0.005181 | 0.6218 | MOT |
| rs7005873 | A | 0.74 | . | *CHD7* | intronic | -4.68 | -7.972 – -1.388 | 0.006735 | 0.6218 | MOT |
| rs27100 | T | 0.43 | . | *TRIO* | intronic | -4.171 | -7.182– -1.161 | 0.008161 | 0.6218 | MOT |
| rs7005873 | A | 0.74 | . | *CHD7* | intronic | -6.41 | -10.54 – -2.279 | 0.00323 | 0.5602 | RRB |
| rs1805482 | A | 0.35 | NM_000834 | *GRIN2B* | p.S555S | 6.074 | 1.944 – 10.2 | 0.005112 | 0.5602 | RRB |
| rs112318565 | G | 0.06 | . | *ARID1B* | intronic | 12.3 | 3.682 – 20.91 | 0.006502 | 0.5602 | RRB |
| rs7844902 | G | 0.72 | . | *CHD7* | intronic | -5.388 | -9.483 – -1.294 | 0.01186 | 0.5602 | RRB |
| rs5891777 | TGGACT | 0.74 | . | *CHD7* | intronic | -5.144 | -9.263 – -1.025 | 0.01665 | 0.5602 | RRB |
| ^a^ “.” Refers to information that is not applicable | | | | | | | | | | |
