# Supplementary material for: Exome sequencing of 85 Williams–Beuren syndrome cases rules out coding variation as a major contributor to remaining variance in social behavior
Source: Mol Genet Genomic Med. 2018 Jul 15;6(5):749–65. doi: 10.1002/mgg3.429 (PMC6160704; doi:10.1002/mgg3.429)
Supplement: Supplementary file 7 [file MGG3-6-749-s007.docx]

| SNP | Alt allele | MAF | Transcript^a^ | Gene | Consequence | Beta | 95% Confidence interval | Raw  p-value | FDR | SRS sub category |
| --- | --- | --- | --- | --- | --- | --- | --- | --- | --- | --- |
| rs35430620 | T | 0.79 | . | *PCTP* | intronic | 9.39 | 5.767 – 13.01 | 2.57E-06 | 0.1711 | AWR |
| rs3803300 | C | 0.84 | NM_001137601 | *ZBTB42* | UTR3 | -11.08 | -15.71 – -6.448 | 1.20E-05 | 0.3714 | AWR |
| Var-6-31322340 | A | 0.07 | . | *HLA-B* | intronic | -12.76 | -18.21 – -7.318 | 1.67E-05 | 0.3714 | AWR |
| rs1804020 | A | 0.27 | NM_001014972 | *ZFN638* | p.V1726M | -8.316 | -12.03 – -4.604 | 3.55E-05 | 0.4874 | AWR |
| rs2960061 | C | 0.85 | . | *PCTP* | intronic | 10.29 | 5.69 – 14.9 | 3.66E-05 | 0.4874 | AWR |
| rs527221 | C | 0.11 | NM_001288765 | *DMPK* | p.L334V | 16.4 | 10.69 – 22.12 | 2.94E-07 | 0.01959 | COG |
| rs572634 | C | 0.11 | . | *DMPK* | intronic | 14.76 | 9.043 – 20.48 | 2.80E-06 | 0.09309 | COG |
| rs2292288 | G | 0.43 | unknown | *SYNM* | unknown | -8.746 | -12.5 – -4.996 | 1.81E-05 | 0.4029 | COG |
| rs2305914 | T | 0.08 | . | *WBP2* | intronic | -15.78 | -22.86 – -8.704 | 3.85E-05 | 0.6403 | COG |
| rs1064512 | C | 0.08 | NM_003038 | *SLC1A4* | p.G37R | 13.12 | 7.061 – 19.18 | 6.14E-05 | 0.8187 | COG |
| rs2076404 | A | 0.69 | . | *TGM6* | intronic | -8.695 | -12.35 – -5.038 | 1.30E-05 | 0.4552 | COM |
| rs2546028 | C | 0.55 | NM_175872 | *ZNF792* | UTR5 | -6.561 | -9.394 – -3.728 | 2.05E-05 | 0.4552 | COM |
| rs2546029 | G | 0.55 | NM_175872 | *ZNF792* | UTR5 | -6.561 | -9.394 – -3.728 | 2.05E-05 | 0.4552 | COM |
| rs491873 | T | 0.59 | . | *TUBA3C* | intronic | -7.645 | -11.03 – -4.258 | 3.16E-05 | 0.5256 | COM |
| rs1811 | G | 0.46 | NM_001099437 | *ZNF30* | p.Q124R | 6.464 | 3.404 – 9.524 | 8.81E-05 | 0.734 | COM |
| rs2651080 | C | 0.31 | NM_175872 | *ZNF792* | p.T333T | 8.116 | 5.113 – 11.12 | 1.09E-06 | 0.02169 | MOT |
| rs1345658 | A | 0.46 | NM_001099437 | *ZNF30* | p.R380K | 6.535 | 4.07 – 9.001 | 1.63E-06 | 0.02169 | MOT |
| rs1811 | G | 0.46 | NM_001099437 | *ZNF30* | p.Q124R | 6.535 | 4.07 – 9.001 | 1.63E-06 | 0.02169 | MOT |
| rs2651079 | T | 0.46 | NM_175872 | *ZNF792* | p.R177Q | 6.535 | 4.07 – 9.001 | 1.63E-06 | 0.02169 | MOT |
| rs2651109 | C | 0.46 | NM_001099437 | *ZNF30* | p.S215S | 6.535 | 4.07 – 9.001 | 1.63E-06 | 0.02169 | MOT |
| rs2546028 | C | 0.55 | NM_175872 | *ZNF792* | UTR5 | -6.773 | -9.831 – -3.715 | 4.26E-05 | 0.5321 | RRB |
| rs2546029 | G | 0.55 | NM_175872 | *ZNF792* | UTR5 | -6.773 | -9.831 – -3.715 | 4.26E-05 | 0.5321 | RRB |
| rs2059404 | A | 0.58 | . | *ARID2* | intronic | -8.358 | -12.16 – -4.558 | 4.75E-05 | 0.5321 | RRB |
| rs7315731 | T | 0.42 | NM_004719 | *SCAF11* | p.V627I | -8.358 | -12.16 – -4.558 | 4.75E-05 | 0.5321 | RRB |
| rs13044892 | A | 0.06 | . | *ATP9A* | intronic | -16.79 | -24.58 – -9.009 | 6.57E-05 | 0.5321 | RRB |
| ^a^ “.” Refers to information that is not applicable | | | | | | | | | | |
